# Observation and enhancement through alkali metal doping of p-type conductivity in the layered oxyselenides Sr_2_ZnO_2_Cu_2_Se_2_ and Ba_2_Zn_1−*x*_O_2−*x*_Cu_2_Se_2_†

**DOI:** 10.1039/d4tc02458c

**Published:** 2024-09-19

**Authors:** Zahida Malik, Sarah Broadley, Sebastian J. C. Herkelrath, Daniel W. Newbrook, Liam Kemp, George Rutt, Zoltán A. Gál, Jack N. Blandy, Joke Hadermann, Daniel W. Davies, Robert D. Smyth, David O. Scanlon, Ruomeng Huang, Simon J. Clarke, Geoffrey Hyett

**Affiliations:** a School of Chemistry, Faculty of Engineering and Physical Sciences, Highfield Campus, University of Southampton Southampton SO17 1BJ UK g.hyett@soton.ac.uk; b Department of Chemistry, University of Oxford, Inorganic Chemistry Lab South Parks Road Oxford OX1 3QR UK; c School of Electronics and Computer Science, University of Southampton Southampton SO17 1BJ UK; d Electron Microscopy for Materials Science (EMAT), University of Antwerp Groenenborgerlaan, 171 B2020 Antwerp Belgium; e Department of Chemistry, University College London 20 Gordon Street London WC1H 0AJ UK; f School of Chemistry, University of Birmingham Edgbaston Birmingham B15 2TT UK

## Abstract

The optoelectronic properties of two layered copper oxyselenide compounds, with nominal composition Sr_2_ZnO_2_Cu_2_Se_2_ and Ba_2_ZnO_2_Cu_2_Se_2_, have been investigated to determine their suitability as p-type conductors. The structure, band gaps and electrical conductivity of pristine and alkali-metal-doped samples have been determined. We find that the strontium-containing compound, Sr_2_ZnO_2_Cu_2_Se_2_, adopts the expected tetragonal *Sr*_*2*_*Mn*_*3*_*SbO*_*2*_ structure with *I*4/*mmm* symmetry, and has a band gap of 2.16 eV, and a room temperature conductivity of 4.8 × 10^−1^ S cm^−1^. The conductivity of the compound could be increased to 4.2 S cm^−1^ when sodium doped to a nominal composition of Na_0.1_Sr_1.9_ZnO_2_Cu_2_Se_2_. In contrast, the barium containing material was found to have a small zinc oxide deficiency, with a sample dependent compositional range of Ba_2_Zn_1−*x*_O_2−*x*_Cu_2_Se_2_ where 0.01 < *x* < 0.06, as determined by single crystal X-ray diffraction and powder neutron diffraction. The barium-containing structure could also be modelled using the tetragonal *I*4/*mmm* structure, but significant elongation of the oxygen displacement ellipsoid along the Zn–O bonds in the average structure was observed. This indicated that the oxide ion position was better modelled as a disordered split site with a displacement to change the local zinc coordination from square planar to linear. Electron diffraction data confirmed that the oxide site in Ba_2_Zn_1−*x*_O_2−*x*_Cu_2_Se_2_ does not adopt a long range ordered arrangement, but also that the idealised *I*4/*mmm* structure with an unsplit oxide site was not consistent with the extra reflections observed in the electron diffractograms. The band gap and conductivity of Ba_2_Zn_1−*x*_O_2−*x*_Cu_2_Se_2_ were determined to be 2.22 eV and 2.0 × 10^−3^ S cm^−1^ respectively. The conductivity could be increased to 1.5 × 10^−1^ S cm^−1^ with potassium doping in K_0.1_Ba_1.9_Zn_1−*x*_O_2−*x*_Cu_2_Se_2_. Hall measurements confirmed that both materials were p-type conductors with holes as the dominant charge carriers.

## Abstract 2

The optoelectronic properties of two layered copper oxyselenide compounds, with nominal composition Sr_2_ZnO_2_Cu_2_Se_2_ and Ba_2_ZnO_2_Cu_2_Se_2_, have been investigated to determine their suitability as p-type conductors.

## Graphical abstract

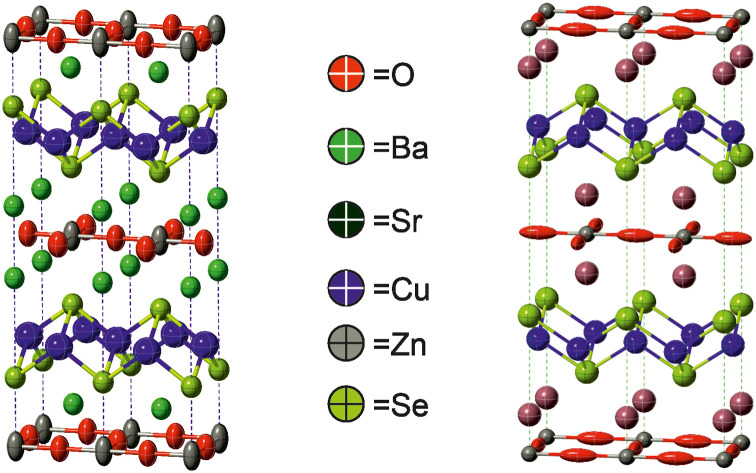

## Introduction

Transparent conductors are a small but significant class of material with a host of commercial applications,^1–4^ with the most widely utilised being the n-type materials indium tin oxide (ITO) and fluorine-doped tin oxide (FTO).^5,6^ The pursuit of new transparent conductors remains a key research problem, in particular identification of a p-type transparent conductor with a low effective hole mass which can be degeneratively doped, as currently none are known that can achieve sufficient levels of both conductivity and transparency.^7–11^ Layered copper(i) oxychalcogenides have been investigated as possible candidates, as the Cu 3d orbitals hybridise with the diffuse chalcogenide p orbitals to give a highly dispersed valence band edge with good hole mobility, while the separation of these copper chalcogenide layers by closed–shell metal oxide layers generates a wide band gap, reducing visible light absorption.^12–14^ For example, strontium-doped LaOCuS has a conductivity of 2.6 × 10^−1^ S cm^−1^, while more complex layered materials Sr_2_GaO_3_CuS and Sr_2_ZnO_2_Cu_2_S_2_ have lower but still significant conductivities of 0.2 × 10^−1^ S cm^−1^ and 1.2 × 10^−1^ S cm^−1^, when sodium doped.^15,16^ All have band gaps in excess of 2.4 eV. In the latter case of Sr_2_ZnO_2_Cu_2_S_2_, the compound was found to have a good hole mobility of 0.74 cm^2^ V^−1^ s^−1^, but with the overall conductivity limited by a low hole yield from the dopant of only 0.3%. All three of these p-type conductors share an isostructural sulfide layer responsible for their conductivity, and in this paper we will consider a pair of materials adopting the *Sr*_*2*_*Mn*_*3*_*Sb*_*2*_*O*_*2*_ structure that contain a similar structural motif, but with copper selenide layers replacing the copper sulfide layers. The size and chemical differences between the sulfide and selenide ion is smaller than between oxide and sulfide ions, so this class of compound can readily accommodate this exchange.

The *Sr*_2_*Mn*_3_*Sb*_2_*O*_2_ structure is comprised of a ‘heavy-anion’ layer adopting a fragment of the *anti-litharge* structure separated by a thin oxide layer which may be described as a single layer fragment of the *perovskite* structure (the ‘light-anion’ layer). These two layers are commonly highlighted, as we have done in this work, by a technically incorrect but useful formulation, 
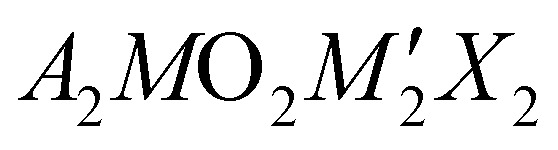
 where *A* is typically Sr or Ba, *M* and *M*′ are transition metals and *X* is a heavy anion, with the two layers being the light anion containing [*A*_2_*M*O_2_]^2+^ and heavy anion containing 
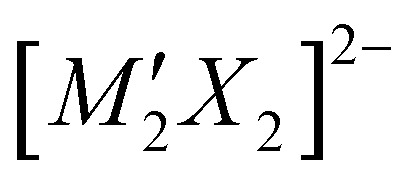
. The earliest examples of this structure type are quaternary materials, such as Sr_2_Mn_3_Sb_2_O_2_ = Sr_2_MnO_2_Mn_2_Sb_2_, where both transition metal sites are occupied by the same metal ion.^17^ The first examples of quinary materials, with different transition metal ions in the heavy anion and light anion layers, were Sr_2_MnO_2_Cu_1.5_S_2_ and Sr_2_ZnO_2_Cu_2_S_2_, both with the more chalcophilic copper found exclusively in the heavy-anion layer bound to sulfur.^18^ These two compounds highlighted the possibility for site segregation of transition metals based on their different characteristics, in this case with the ‘softer’ copper associating with sulfur, while the ‘harder’ zinc or manganese ions are found in the oxide layer. Sr_2_MnO_2_Cu_1.5_S_2_ and Sr_2_ZnO_2_Cu_2_S_2_ were first identified in 1997, and since then over 40 unique phases have been found to adopt the *Sr*_*2*_*Mn*_*3*_*Sb*_*2*_*O*_*2*_ structure type, but the most common are those with copper chalcogenide heavy-anion layers.^17,19–36^

The majority of the *Sr*_*2*_*Mn*_*3*_*Sb*_*2*_*O*_*2*_ phases so far identified adopt a tetragonal space group with a square planar geometry for the transition metal in the oxide layer. The first exception to this was identified by Clarke *et al.* when investigating Ba_2_ZnO_2_Ag_2_Se_2_.^23^ The compound was initially interpreted – based on X-ray diffraction (XRD) – to have adopted the standard *I*4/*mmm* structure. However, in this phase the long Ag–Se bond in the anti-litharge layer and large barium in the oxide layer expand the unit cell such that the zinc square planar site would require extremely elongated Zn–O bonds of 2.14 Å. This can be compared to the much shorter bond lengths of 2.00 Å found in Sr_2_ZnO_2_Cu_2_S_2_. A more detailed investigation of the Ba_2_ZnO_2_Ag_2_Se_2_ structure using neutron powder diffraction (NPD), electron diffraction (ED) and the local probe extended X-ray absorption fine structure (EXAFS) revealed that the zinc environment undergoes a distortion to produce linear units, relieving the apparent tension of the four long zinc oxide bonds with two short bonds (1.91 Å) and two very long interactions (2.37 Å) instead. This occurs through a long range ordered shift in the oxide ion site position and a reduction in symmetry to a larger *Cmca* cell.^23^ However, as the symmetry reduction and change in zinc environment identified in Ba_2_ZnO_2_Ag_2_Se_2_ is ultimately caused by only a small shift in the oxide ion position, it is challenging to identify based on X-ray diffraction data alone. This begs the question, have other *Sr*_*2*_*Mn*_*3*_*Sb*_*2*_*O*_*2*_ materials been incorrectly assigned as tetragonal? A useful indicator is the bond valance sum (BVS) of the square planar metal site. If Ba_2_ZnO_2_Ag_2_Se_2_ is modelled as tetragonal, an extremely low BVS of +1.35 is found for zinc, but after the symmetry reduction a more satisfactory BVS value of +1.63 can be determined instead. Thus, a low BVS could be used as an indicator of the possibility of the orthorhombic distortion in compounds isostructural to Sr_2_ZnO_2_Cu_2_S_2_.

This question was also considered by Chen *et al.* for the cobalt and manganese containing silver selenide analogues, Ba_2_CoO_2_Ag_2_Se_2_ and Ba_2_MnO_2_Ag_2_Se_2_, where the conventional high symmetry tetragonal structure was confirmed by electron diffraction, as the slightly larger cobalt and manganese ions were able to maintain square planar geometry with BVS values of +1.42 and +1.78, although modelling suggested that the cobalt was on the cusp of stability between the *I*4/*mmm* and *Cmca* structures.^29^ More recently the silver telluride Ba_2_ZnO_2_Cu_2_Te_2_ has been identified through high pressure synthesis. At room temperature this unambiguously adopts the orthorhombic form, with a BVS for zinc in the linear environment of +1.73. Without the distortion the square planar model would have a BVS of only +1.12,^36^ a significant level of underbonding. However, upon heating Ba_2_ZnO_2_Cu_2_Te_2_, a phase transition to *I*4/*mmm* symmetry is observed, which is interpreted not as conversion to a square planar zinc environment, but a disordered arrangement of neighbouring linear zinc environment in either *cis* or *trans* alignment, rather than the uniform *trans* arrangement seen at room temperature – providing a potential third arrangement of the transition metal geometry to consider in this structure type.

In this paper we report the structural characterisation of two new layered oxyselenides, with nominal composition Sr_2_ZnO_2_Cu_2_Se_2_ and Ba_2_ZnO_2_Cu_2_Se_2_, having determined their structures from X-ray and neutron diffraction measurements, finding that the strontium-containing material adopts the expected tetragonal symmetry, but that the barium-containing compound adopts the disordered high-temperature Ba_2_ZnO_2_Cu_2_Te_2_ structure with a linear zinc geometry. We have also investigated the optoelectronic properties and the effect of sodium and potassium doping in both phases. We will show that both are p-type conductors, and that the conductivity can be enhanced through appropriate alkali metal doping, and that sodium-doped Sr_2_ZnO_2_Cu_2_Se_2_ is of particular interest as a wide band gap p-type conductor with a high concentration of charge carriers.

## Experimental methods

### Synthesis

Initial powders of Sr_2_ZnO_2_Cu_2_Se_2_ and Ba_2_ZnO_2_Cu_2_Se_2_ were prepared from a mixture of ZnSe (Alfa Aesar, 99.99%), Cu_2_Se (Alfa Aesar, 99.5%) and either SrO (Aldrich, 99.99%) or BaO (Aldrich, 99.99%) in a 1 : 1 : 2 ratio to give a total mass of approximately 0.5 g. The precursor mixture was ground, loaded into alumina crucibles and sealed under vacuum in fused silica tubes before being heated to 600 °C at 1 °C min^−1^ and kept at this temperature for 12 h. After this, the ampoules were broken inside a N_2_-filled glove box, and samples pulverized before cold pressing into pellets under 10 tonnes of pressure. The pellets were resealed into silica tubes and annealed at 700 °C for 24 h. To get good quality crystalline materials for the single crystal studies, Ba_2_ZnO_2_Cu_2_Se_2_ and Sr_2_ZnO_2_Cu_2_Se_2_ pellets were heated at 0.1 °C min^−1^ to 900 °C and 930 °C respectively, and kept for 6 h at these temperatures, before cooling to room temperature at 0.1 °C min^−1^.

Larger 4 g samples for NPD measurements were prepared using a similar procedure from Se (Alfa Aesar 99.999%), Zn (Alfa Aesar Puratronic 99.998%), Cu (99.995%, Alfa Aesar) and BaO (99.99% Sigma Aldrich) or SrO, heated at 920 °C for 24 h. For the neutron diffraction sample, the SrO precursor was not purchased but instead prepared from thermal decomposition of SrCO_3_ (Alfa Aesar, 99.995%) by heating for 18 h at 850 °C and for a further 6 h at 1100 °C under dynamic vacuum.

For the samples doped with alkali metals, with nominal composition *A*_*x*_Sr_2−*x*_ZnO_2_Cu_2_Se_2_ and *A*_*x*_Ba_2−*x*_ZnO_2_Cu_2_Se_2_ where *A* = Na or K and *x* = 0.05, 0.10 and 0.33, a similar procedure was adopted as above, but with a precursor mix of *A*_2_Se, *A*O, Cu_2_Se, ZnO and Zn in the appropriate ratio. Due to the relatively low melting point of zinc, a staged heating cycle was used with a ramp rate of 1 °C min^−1^, initially to 80 °C with an 8 h dwell, followed by a ramp to 400 °C with a 12 h dwell, then 600 °C held for 12 h, before a final anneal at 700 °C for 24 h. The reagents used were supplied as above, but with the addition of ZnO (Sigma Aldrich, 99.99%) and Zn (Sigma Aldrich, 99.9%). Na_2_Se and K_2_Se were synthesised by direct reaction of the elements in liquid ammonia using Se (Sigma Aldrich, 99.99%) and surface cleaned Na (Merck 99.9%) or K (Merck 98%).^37^

### Structural characterisation

The structures of the materials were initially identified by Rietveld refinement against powder X-ray diffraction data collected in the Bragg–Brentano geometry over the range of 10° < 2*θ* < 100° with a collection time of 12 hours using a Bruker D2 PHASER diffractometer equipped with a copper X-ray source. Rietveld refinement was carried out using the GSAS-II software package.^38^ For the structural model, the lattice parameters, atomic site and isotropic thermal displacement parameters were refined. The sample broadening effect on the peak profile was accounted for by refinement of microstrain and particle size parameters. The instrumental peak broadening was fixed using a pseudo-Voigt function which had been modelled in a refinement against a LaB_6_ standard in a prior experiment.

Neutron powder diffraction measurements were carried out on 3–4 g samples of Sr_2_ZnO_2_Cu_2_Se_2_ and Ba_2_ZnO_2_Cu_2_Se_2_ using the time of flight neutron diffractometer POLARIS at ISIS, Didcot, UK with data banks at 2*θ* positions of 35°, 90° and 145° (pre-2011 configuration).^39^ Refinement against PND was conducted using the TOPAS Academic version 5 software.^40^ These measurements allowed for more accurate determination of the oxide ion position in the structures, and were supported by electron diffraction measurements carried out on a Phillips CM20 transmission electron microscope with a LaB_6_ filament.

The final structural characterisation method used was single crystal X-ray diffraction. Suitable crystals of Ba_2_ZnO_2_Cu_2_Se_2_ and Sr_2_ZnO_2_Cu_2_Se_2_ were mounted on the tip of a sample holder in oil, and diffraction patterns collected on a Synergy Custom system, HyPix diffractometer. The crystals were kept at a steady temperature of 100 K during data collection using a cryogenic nitrogen gas flow. The structures were solved with the ShelXT 2014/5 program,^41^ using dual-space methods in the Olex2 graphical interface. The models were refined with ShelXL 2016/6 using full matrix least squares minimisation on *F*^2^.^42^

### Optoelectronic properties

Optical properties were determined using diffuse reflectance spectroscopy recorded over the UV-vis range of 300–900 nm with a PerkinElmer Lambda 750S instrument, equipped with an integrating sphere. The reflectance data sets were used to determine the optical band gaps of the materials employing the method outlined by Poeppelmeier *et al.*^43^

Room temperature conductivity measurements were carried out on both undoped and doped samples. The materials were pressed under 2 tons of pressure in an 8 mm diameter die to produce pellets with a thickness of between 1 mm and 2 mm. The pellets were then annealed in an alumina crucible under vacuum in a sealed silica tube at 700 °C for 12 h. A conducting silver paste (MG Chemicals, Epoxy, 2 W m^−1^ K^−1^, 8.3 N mm^−2^) was applied at the four points opposite to one another on the perimeter of the sample pellets, then connected to a Keithley DMM6500 digital multimeter, using an Ecopia SPCB-01 four-point probe spring clip mounting board. The van der Pauw method was used to determine the average resistance of the sample.^44^

Further characterisation of the electronic properties of the pellets were carried out using a Nanometrics HL5500 Hall effect system with a van der Pauw configuration. A magnetic field of 0.5 T was used with the Hall measurement approach to determine conductivity, carrier mobility and carrier density at room temperature. *I*–*V* curves were recorded before each measurement to ensure Ohmic conduction as well as to optimize current for maximized voltage signal (20 mV). Room temperature Seebeck coefficients were measured using a JouleYacht thermoelectric parameter test system (MRS-3L) and were determined using the differential method with a maximum temperature difference of 10 K.

### Computational studies

Calculations were carried out using the Vienna Ab initio Simulation Package (VASP),^45,46^ relaxed using the PBEsol functional^47^ with a Hubbard-like *U* correction of 5.17 eV on Cu.^48–55^ The projector-augmented wave (PAW) method was used to describe interactions between the core and valence electrons.^56^ A plane-wave cut-off of 550 eV was used to avoid Pulay stress^57^ and a *Γ*-centred mesh with a *k*-point density of at least 70 Å^3^ was used to sample the Brillouin zone. Net forces on ions were reduced to less than 0.01 eV Å^−1^. The screened hybrid exchange correlation functional, HSE0644,^58^ has been shown in previous work to accurately calculate the electronic structure of this family of compounds,^13^ so was used here to calculate band structures. Band structures were produced using the Sumo python package.^59^ Charge carrier effective masses were calculated using the curvature and electronic band extrema.

Competing phases for Sr_2_ZnO_2_Cu_2_Se_2_ and Ba_2_ZnO_2_Cu_2_Se_2_ compounds were identified using the Materials Project API,^60^ limiting the search to those compounds that appear in the ICSD. These competing phases were relaxed using the same calculation parameters described above, with a denser *k*-point mesh of at least 100 Å^3^ used for metallic phases. All PBEsol+*U* calculations were carried out using the Fireworks python package^61^ and final values for energy above the convex hull of the compositional phase diagram (*E*_hull_) were calculated using the Pymatgen python package.^62^

## Results and discussion

### Synthesis and structure of pristine Sr_2_ZnO_2_Cu_2_Se_2_ and Ba_2_ZnO_2_Cu_2_Se_2_

**Fig. 1:**
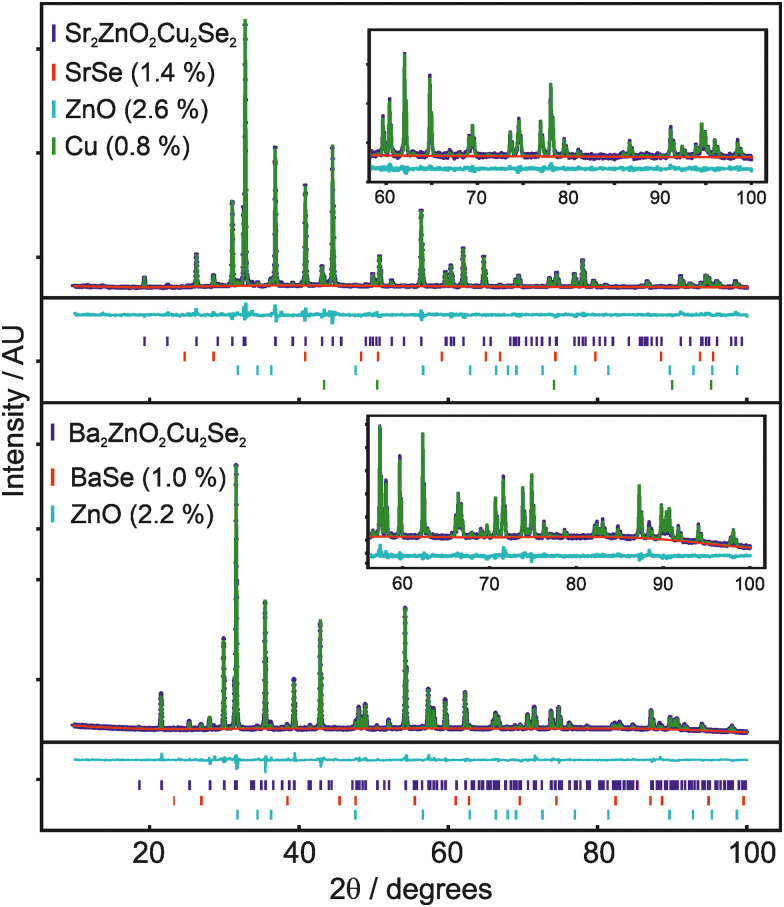
Rietveld refinement against powder X-ray diffraction data, showing data in blue, background in red, model pattern in blue and difference curve in cyan. Insert shows a 60° to 100° 2*θ* expanded region to highlight the high angle fit. Tick marks below the pattern are for the main phase, BaSe or SrSe, ZnO and Cu, where present. Upper panel shows X-ray pattern and model fit for nominal composition Sr_2_ZnO_2_Cu_2_Se_2_, lower panel for Ba_2_ZnO_2_Cu_2_Se_2_.

**Fig. 2:**
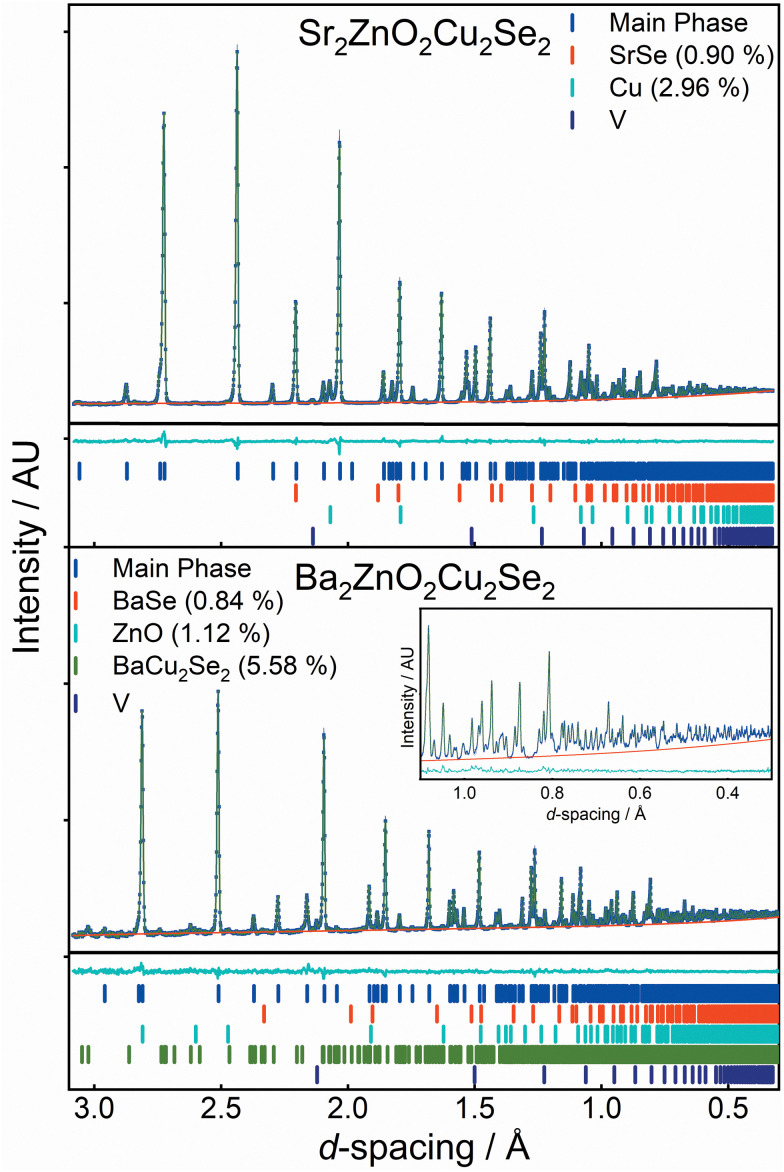
Rietveld refinement against neutron diffraction data. Upper image shows neutron pattern and model fit for nominal composition Sr_2_ZnO_2_Cu_2_Se_2_ at room temperature from bank C of POLARIS. The lower panel shows data for Ba_2_ZnO_2_Cu_2_Se_2_ at 2 K also from bank C of POLARIS. In each figure the data is in blue, background in red, model pattern in green and difference curve in cyan. The expanded region insert shows the 0.3 Å to 1.1 Å data to highlight the low *d*-spacing fit. Tick marks below the pattern are for the main phase, BaSe or SrSe, ZnO, Cu and BaCu_2_Se_2_, where present. Vanadium contribution is from the sample environment.

The structures of both Sr_2_ZnO_2_Cu_2_Se_2_ and Ba_2_ZnO_2_Cu_2_Se_2_ were confirmed using a combination of powder X-ray diffraction (Fig. 1), powder neutron diffraction (Fig. 2), and single crystal X-ray diffraction. Aggregating the results of these techniques, we find that both compounds adopt the *Sr*_*2*_*Mn*_*3*_*Sb*_*2*_*O*_*2*_ structure type, with the tetragonal *I*4/*mmm* archetype enabling satisfactory modelling of the data for both compounds, although for Ba_2_ZnO_2_Cu_2_Se_2_ there is evidence of a local reduction in symmetry, and a better fit is found with a linear zinc geometry modelled with a disordered split site oxygen position. This is consistent with the appearance of extra features in the electron diffraction patterns that would not be observed with the idealised *I*4/*mmm* model with an unsplit oxygen site. The structural observations are further supported by DFT calculations which find that Sr_2_ZnO_2_Cu_2_Se_2_ is stable relative to any competing phases, *i.e.* an energy above hull (*E*_hull_) of 0 eV, while Ba_2_ZnO_2_Cu_2_Se_2_ when modelled with the *I*4/*mmm* structure, has an *E*_hull_ of 0.03 eV, indicating slight energetic instability, as the model cannot effectively account for the localised distortion of the zinc geometry.

The powder X-ray diffraction data collected from the sample of Sr_2_ZnO_2_Cu_2_Se_2_ was initially modelled assuming the *I*4/*mmm Sr*_*2*_*Mn*_*3*_*Sb*_*2*_*O*_*2*_ structure type previously found to be adopted by Sr_2_ZnO_2_Cu_2_S_2_, the sulfide containing analogue.^18^ After refinement, this model was found to give a good fit to the data, with an overall *Rw*_p_ of 4.54% and a sample purity of 95.2 wt%. The refined lattice parameters were found to be *a* = 4.06703(4) Å and *c* = 18.3849(2) Å, and the isotropic displacement parameters for all the atoms were reasonable, covering a range between 0.017 Å^2^ and 0.029 Å^2^. The remaining Bragg peaks in the pattern could be matched to SrSe (1.4 wt%), ZnO (2.6 wt%) and Cu (0.8 wt%). The square planar zinc environment in Sr_2_ZnO_2_Cu_2_Se_2_ was found to have Zn–O bond lengths of 2.03 Å, yielding a BVS of +1.83; sufficiently close to the ideal +2.0 that we would not expect the orthorhombic distortion to occur. This was supported by single crystal X-ray diffraction data collected at 100 K which could also be effectively modelled using the *I*4/*mmm* structure, with fit parameters of 4.28% and 2.91% for w*R*_2_ and R_1_, and with a residual electron density peak of 1.4 e Å^−3^ and hole of −0.9 e Å^−3^. The thermal ellipsoids were modelled using anisotropic parameters, and for most of the atoms these showed broadly spherical displacements, with the exception of the zinc and oxide ions which showed a small prolate distortion in the *z*-axis direction. Crucially, the oxide ions do not show any extensive distortion in the basal plane that would be characteristic of an underlying orthorhombic distortion or shift towards a linear zinc environment. An attempt was made to refine the zinc and oxide ion occupancy in the single crystal data model, but no evidence of any vacancy was found, with both occupancies refining to unphysical values slightly above 1, and so both were set to equal 1 in the final refinement. An image of the unit cell derived from the single crystal data refinement, showing the atom displacements, can be found in the ESI.† Refinement against TOF neutron diffraction data, similarly, was well fit using the *I*4/*mmm* model with a *Rw*_p_*o*f 3.40%, and without any unexpected elongation of the anisotropic displacement ellipsoids. The sample purity was found to be 96.1%, with SrSe (0.9%) and Cu (3.0%) found alongside the main phase. As neutron data is more sensitive to light atoms, all atoms could be refined with anisotropic displacement parameters. An attempt was made to refine the site occupancy factors, but again these were all found to remain fully occupied, within the error of the measurement. Both the room temperature NPD and XRD powder refinements gave, with less than 0.25% difference, the same bond lengths. Overall, we are confident to assign the conventional, undistorted *I*4/*mmm* structure to Sr_2_ZnO_2_Cu_2_Se_2_. Full details of the refinements can be found in the ESI,† but a summary is provided in Table 1.

**Table 1:** Comparison of the results of Rietveld refinement against single crystal and powder X-ray diffraction data, and neutron powder diffraction data, for Sr_2_ZnO_2_Cu_2_Se_2_. Data provided is the experimental temperature, sample colour, space group and lattice parameters, sample purity, fractional co-ordinates of strontium and selenium (atoms not on special sites) and key bond lengths and angles

Measurement	PXRD, 4444 data points, *Rw*_p_ = 4.63%	SXRD, 4153 reflections, GoF = 1.09, w*R*_2_ = 4.28%, *R*_1_ = 2.91%	NPD, *Rw*_p_ = 3.40%
Temp.	298 K	100 K	298(2) K
Colour	Red	Rose pink	Red
Space group	*I*4/*mmm*	*I*4/*mmm*	*I*4/*mmm*
*a* (Å)	4.06703(4)	4.0562(2)	4.06518(4)
*c* (Å)	18.3849(2)	18.2727(15)	18.3751(2)
Volume (Å^3^)	304.099(7)	300.64(4)	303.661(8)
Purity (wt%)	95.2	N/A	96.1
Sr (0.5, 0.5, *z*)	0.08771(6)	0.08807(3)	0.08796(3)
Se (0, 0, *z*)	0.16790(7)	0.16865(4)	0.16853(2)
Zn–O (Å)	2.03352(2)	2.0281(1)	2.03259(2)
Zn–Se (Å)	3.087(1)	3.0817(8)	3.0968(4)
Cu–Se (Å)	2.5325(8)	2.5145(5)	2.5244(3)
*α*, Se–Cu–Se (°)	106.83(4)	107.52(3)	107.26(1)
Sr–O (Å)	2.5953(7)	2.5890(4)	2.5969(3)
Sr–Se (Å)	3.2317(8)	3.2240(4)	3.2334(4)

For the barium-containing sample of nominal composition Ba_2_ZnO_2_Cu_2_Se_2_, the picture is more complex. The X-ray diffraction data collected on these samples, both single crystal and powder, were initially modelled assuming the *I*4/*mmm* structure, and this model provided a reasonable fit to both datasets. The Rietveld refinement against the powder data set produced a fit with a *Rw*_p_ of 3.86%, with a main phase sample purity of 96.8 wt% with small amounts of BaSe (1.0%) and ZnO (2.2%) identified from peaks in the pattern. Similarly, the fit to the single crystal X-ray diffraction data with the *I*4/*mmm* model was good with a w*R*_2_ of 2.82%, GoF of 1.11 and residual electron density of −0.93 e Å^−3^ to 0.73 e Å^−3^. The zinc occupancy was refined in both models, and a small deficiency was found in both, 0.3% from the powder diffraction model, and 1.3% from the single crystal – suggesting a small sample dependent deficiency. Despite the good overall fit in the powder model, the value for the isotropic oxide ion displacement was larger than expected at 0.069 Å^2^, compared with a value of 0.022 Å^2^ found in the refined X-ray powder model of Sr_2_ZnO_2_Cu_2_Se_2_. In the single-crystal-XRD-derived model, where anisotropic ellipsoids could be refined, the oxide ion displayed a large elongation towards the zinc ion with the long axis of the oxide ion displacement in a ∼3 : 1 ratio to the other semi-axes. A diagram of this unit cell can be found in the ESI.†

One interpretation of the extended displacement ellipsoid identified for the oxide ion in Ba_2_ZnO_2_Cu_2_Se_2_ is that the structure has undergone a symmetry reduction like that found in Ba_2_ZnO_2_Ag_2_Se_2_, which contains silver selenide layers, with either ordered or disordered displacement of the oxide ions to produce linear [ZnO_2_]^2−^ units. Both of these underlying structures can be approximately modelled using an elongated ellipsoid in the *I*4/*mmm* symmetry. Assuming there is no ion displacement gives a zinc oxide bond length of 2.10 Å, and a BVS value of +1.45, which would indicate a significant under-bonding compared to either Sr_2_ZnO_2_Cu_2_S_2_ or Sr_2_ZnO_2_Cu_2_Se_2_. However, the under-bonding is not as extreme as the BVS value of +1.35 found in the *I*4/*mmm* model for Ba_2_ZnO_2_Ag_2_Se_2_ where the orthorhombic distortion was first identified, but is similar to the value of +1.42 found in Sr_2_CoO_2_Ag_2_Se_2_ which was considered to be near the boundary of the transition between the *I*4/*mmm* and *Cmca* phases.^29,36^ To determine if the symmetry reduction has occurred in Ba_2_ZnO_2_Cu_2_Se_2_ neutron diffraction and electron diffraction measurements were performed.

**Fig. 3:**
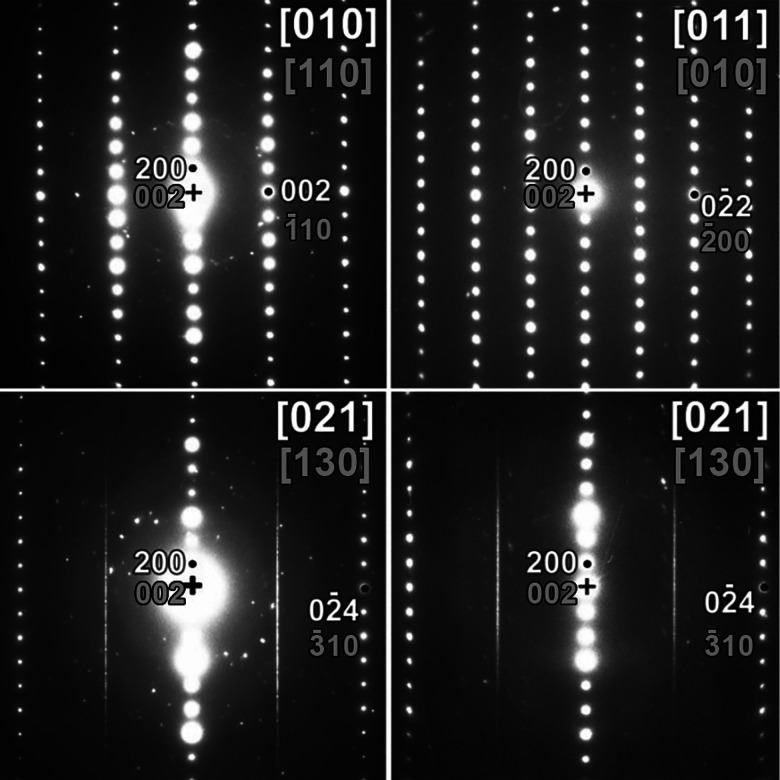
Electron diffraction images collected on a sample of Ba_2_ZnO_2_Cu_2_Se_2_. The white indices are relative to the *Cmca* cell, the gray ones to the *I*4/*mmm* cell. The streaks present in the bottom two electron diffraction patterns can be explained assuming the presence of twinned layers or stacking faults in the *Cmca* cell, but not in the *I*4/*mmm* cell, where there are no lattice points at any positions of these rows. Two different [021] (*Cmca* indexing) patterns are shown to demonstrate the difference in defect structure between different particles, with the left-hand one showing more order as can be seen from the presence of clear reflections within the streaks.

The results of the electron diffraction experiments on a sample of Ba_2_ZnO_2_Cu_2_Se_2_ are shown in Fig. 3. This reveals the presence of diffuse streaks within the [021] zone, assuming indexing in the *Cmca* symmetry. Sharp reflections along these rows would break the tetragonal *I*4/*mmm* symmetry and be strong evidence of a change to orthorhombic (*Cmca*) symmetry. The occurrence of streaks along the c*-axis at only those rows characteristic for *Cmca* indicates that there are stacking faults which disturb the ordered fragmentation of [ZnO_2_]^2−^ layers found in Ba_2_ZnO_2_Ag_2_Se_2_, which results in symmetry lowering, but which do not affect the stacking of the two layers found in the *I*4/*mmm* structure. As there is no long-range order, the *I*4/*mmm* symmetry is appropriate for the average structure, with localised, disordered fragmentation of the [ZnO_2_]^2−^ layers. Therefore, the disordered model found in Ba_2_ZnO_2_Cu_2_Te_2_, above 350 K, should be considered for Ba_2_ZnO_2_Cu_2_Se_2_. However, it should be made clear that a model in the ideal structure in *I*4/*mmm* without the split oxygen site is not compatible with the electron diffraction results, because in that case the streaks shown in Fig. 3 would be absent.

**Fig. 4:**
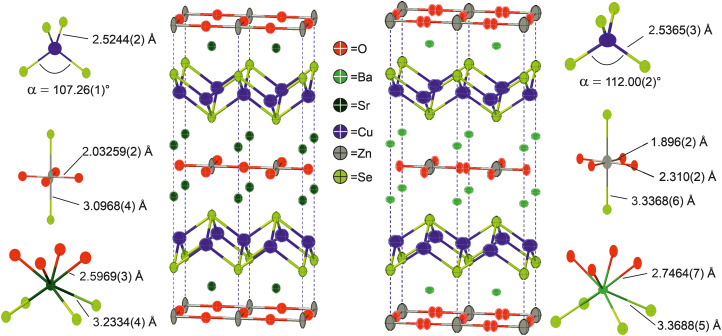
Representations of the structures of Sr_2_ZnO_2_Cu_2_Se_2_ and Ba_2_Zn_1−*x*_O_2−*x*_Cu_2_Se_2_ refined in *I*4/*mmm*, with data taken from refinement of models to room temperature neutron powder diffraction data. Main figures show the Cu–Se and Zn–O bonds. Also shown are diagrams of the Sr, Ba, Zn and Cu environments with selected bond lengths and angles. Note the split oxygen site, indicating disordered arrangement in the zinc oxide layer in Ba_2_Zn_1−*x*_O_2−*x*_Cu_2_Se_2_.

Neutron powder diffraction data were collected at ambient temperature and at 2 K on the larger powder sample of Ba_2_ZnO_2_Cu_2_Se_2_ and modelled in *I*4/*mmm* symmetry using a split oxygen ion site with 50% occupancy – the model most consistent with the electron diffraction results. In this model the atom displacement parameters were refined as anisotropic, except for the oxygen site where the in-plane displacements were constrained to be isotropic. There was no indication of a change of model with temperature. The refinements against these data provided a good fit (4.06% at room temperature, 1.89% at 2 K), and are shown in Fig. 2.‡‡For completeness, the single-site *I*4/*mmm* model (with no split O) and *Cmca* model were also tested, but provided worse fits than the split site model. Details have been provided in the ESI† for comparison. From the refinement it was found that the sample was 92.5% pure, with reasonably large impurities of Ba_2_Cu_2_Se_2_ (5.6%), ZnO (1.1%) and (BaSe 0.8%). Independent refinements of the zinc and oxide ion occupancies were included in the refinements at both temperatures and were consistent with a deficiency of ZnO of 6(1)% giving a refined composition of Ba_2_Zn_0.94(1)_O_1.94(1)_Cu_2_Se_2_, averaged over the two refinements (*i.e.* the Zn and O deficiencies were equal within the uncertainties of the refinements). There was no evidence for deficiency on the Cu site (occupancy refined to 1.000(2)) and thus the refinements did not suggest any electron or hole doping. It is unclear why the oxygen site, and hence the linear zinc oxide units, are disordered, rather than the long-range ordering leading to *Cmca* symmetry observed in Ba_2_ZnO_2_Ag_2_Se_2_, but this may be driven by the zinc and oxygen vacancies in the layer. With the displacement of the oxide ion in this refinement, the average zinc environment is linear with two short Zn–O bonds of 1.90 Å and two longer interactions of 2.31 Å, giving an overall BVS of +1.76, a much more reasonable value than the +1.45 determined initially from the single-site model refinement against powder X-ray diffraction data. Given the success of the split-site model in accounting for both the neutron and electron diffraction data, the single crystal X-ray diffraction data were re-evaluated and modelled with an improved fit using this approach. Detailed results of the refinements against all the diffraction data sets using this model for Ba_2_Zn_1−*x*_O_2−*x*_Cu_2_Se_2_ can be found in Table 2. Representations of the structures of both Ba_2_Zn_1−*x*_O_2−*x*_Cu_2_Se_2_ and Sr_2_ZnO_2_Cu_2_Se_2_ can be found in Fig. 4, with the bond lengths taken from the room temperature neutron diffraction refinements, due to the greater sensitivity of these measurements to the position of the oxide ions.

**Table 2:** Comparison of the results of Rietveld refinement against single crystal X-ray diffraction data, and neutron powder diffraction data, for Ba_2_Zn_1−*x*_O_2−*x*_Cu_2_Se_2_. Data provided is the experimental temperature, sample colour, space group and lattice parameters, sample purity, zinc and oxygen site occupancy, fractional co-ordinates of barium, selenium and oxygen (atoms not on special sites), key bond lengths and angles

Measurement	SXRD, 4682 reflections, GoF = 1.104, w*R*_2_ = 2.78%, *R*_1_ = 1.29%	NPD, *Rw*_p_ = 1.89%	NPD, *Rw*_p_ = 4.06%
Temp.	100 K	2 K	298 K
Colour	Orange	Brown	Brown
Space group	*I*4/*mmm*	*I*4/*mmm*	*I*4/*mmm*
*a* (Å)	4.1871(2)	4.18641(9)	4.20579(9)
*c* (Å)	18.9579(12)	18.9687(4)	19.0207(4)
Volume (Å^3^)	332.37(4)	332.447(16)	336.450(16)
Purity (wt%)	N/A	92.5	92.5
Zn occ.	0.991(4)	0.958(3)	0.940(3)
O occ./0.5	0.4972	0.4874(16)	0.4853(14)
Ba (0.5, 0.5, *z*)	0.40746(2)	0.40735(5)	0.40777(5)
Se (0, 0, *z*)	0.17534(2)	0.17523(3)	0.17543(3)
O (0, *y*, 0)	0.5435(11)	0.5433(4)	0.5492(4)
Zn–O short (Å)	1.911(5)	1.9118(16)	1.8960(16)
Zn–O long (Å)	2.276(5)	2.2746(16)	2.3098(16)
Zn–Se (Å)	3.3241(8)	3.3239(6)	3.3368(6)
Cu–Se (Å)	2.5272(3)	2.5285(3)	2.5365(3)
*α*, Se–Cu–Se (°)	111.873(16)	111.76(2)	112.00(2)
Ba–O (Å)	2.7376(4)	2.7391(6)	2.7464(6)
Ba–Se (Å)	3.3510(2)	3.3492(6)	3.3688(5)

**Fig. 5:**
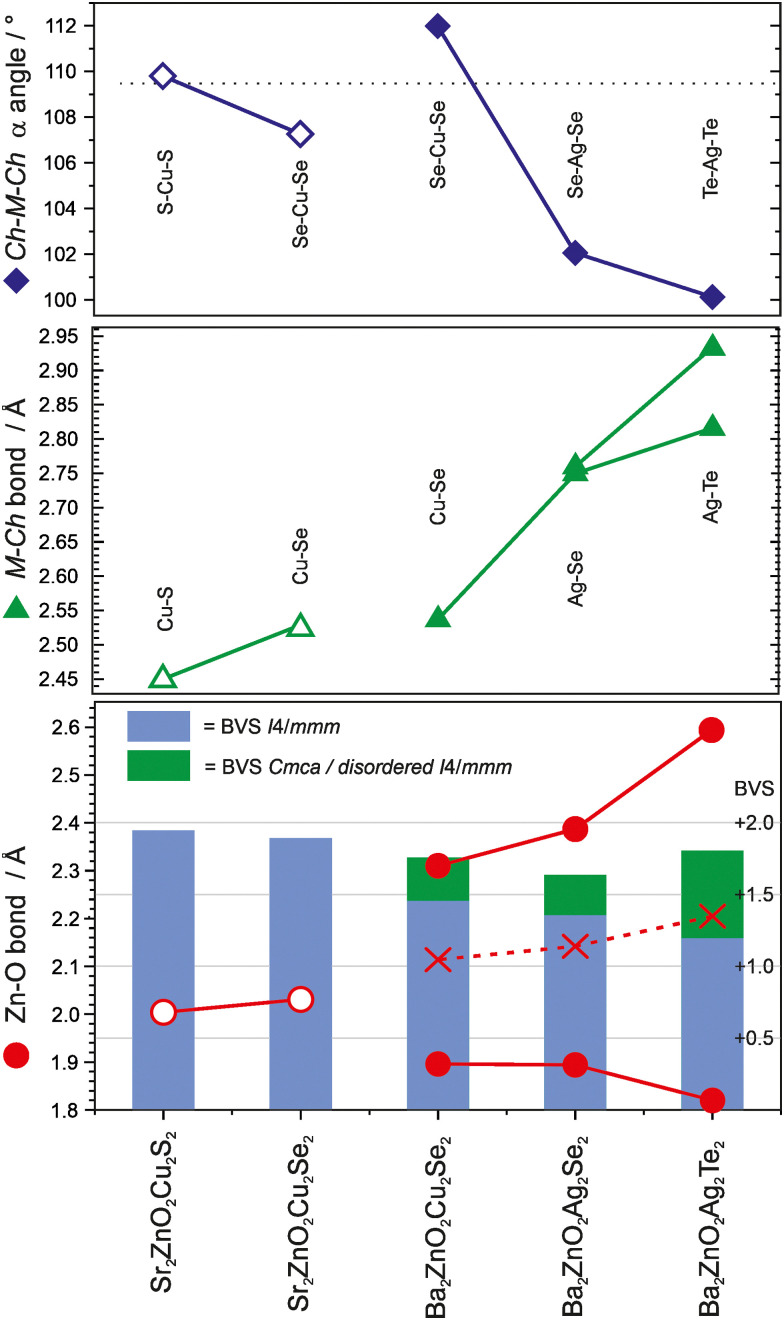
Plot of the key angles and distances in the light and heavy anion layers of the five zinc containing “*Sr*_*2*_*Mn*_*3*_*Sb*_*2*_*O*_*2*_” phases. In the top panel the coinage metal bond angle is shown, and in the middle panel the bond length. The bottom panel show the zinc oxygen bond lengths, with ‘X’ marking the bond length that would be found without the oxygen site displacement and structural distortion. The blue bar indicates the BVS of the zinc site in the square planar geometry, with the green bar indicating the increase in BVS due to the observed switch to a linear environment. Samples containing Sr are shown with open symbols, those with Ba are shown with closed symbols.

The two new compositions reported here can be combined with three related phases reported in the literature to produce a sequence of five compositions with the *Sr*_*2*_*Mn*_*3*_*Sb*_*2*_*O*_*2*_ structure that contain planar zinc oxide layers separated by coinage metal chalcogenide layers and which can be compared for structural trends. In order of increasing *a* lattice parameter length these are Sr_2_ZnO_2_Cu_2_S_2_, Sr_2_ZnO_2_Cu_2_Se_2_, Ba_2_Zn_1−*x*_O_2−*x*_Cu_2_Se_2_, Ba_2_ZnO_2_Ag_2_Se_2_, and Ba_2_ZnO_2_Ag_2_Te_2_. The changes in the *a* lattice parameter across this series are accommodated in the chalcogenide layer through the large variation in the *Ch*–*M*–*Ch* (*M* = Cu, Ag) angle from 100° to 112°, as shown in Fig. 5, indicating significant distortion from the tetrahedral ideal. As established above, for the zinc environment to maintain sufficient bonding there is a transition across the sequence from square planar zinc ions to a linear zinc coordination. For the first two materials in the sequence with strontium on the *A* site, zinc is in the square planar environment with bond lengths of 2.00 Å and 2.03 Å (=*a*/2), and bond valence sums (BVS) of +1.94, and +1.84, respectively. In the final two, silver-containing compounds the expansion of the equivalent zinc–zinc distance to 4.29 Å in Ba_2_ZnO_2_Ag_2_Se_2_ and 4.43 Å in Ba_2_ZnO_2_Ag_2_Te_2_ means the average model (oxygen located on the mean position with an elongated ellipsoid) would give much lower nominal BVS values of +1.35 and +1.12. Instead the orthorhombic distortion to a linear zinc environment is observed through a shift of the oxygen site position to produce two long interactions and two shorter bonds with lengths of 1.90 Å and 1.82 Å in Ba_2_ZnO_2_Ag_2_Se_2_ and Ba_2_ZnO_2_Ag_2_Te_2_ respectively, raising the BVS values to +1.63 and +1.80. In Fig. 5 the Zn–O bond length that would be found in the average position is marked with an ‘×’, and the resulting BVS by the blue bar. The actual bond lengths are shown with circle symbols, and the increase in BVS caused by the shift to the linear arrangement is indicated by the green bar. The remaining compound, Ba_2_Zn_1−*x*_O_2−*x*_Cu_2_Se_2_, sits at the transition of these two symmetry models, with the disordered split site raising the BVS from +1.45 to +1.76. This sequence of compounds shows a key trend in which the overall structure type is maintained despite the substitution of increasingly larger ions (Sr for Ba, Ag for Cu, and Te or Se for S), through distortion of the individual layers – in the heavy anion layer *via* the tetrahedral angle of the coinage metal site and in the light anion layer *via* the transition from a square planar to a linear zinc environment.

This analysis suggests that for *Sr*_*2*_*Mn*_*3*_*Sb*_*2*_*O*_*2*_-structured materials, the minimum BVS for the zinc ion in this structure type is approximately +1.6, and that in compositions where the square planar *I*4/*mmm* structure would reduce the bonding below this value, oxide ion displacement occurs to shorten a pair of the Zn–O distances and increase bond valence. This may occur in a long-range-ordered fashion to yield a structure in *Cmca* as in Ba_2_ZnO_2_Ag_2_Se_2_ and Ba_2_ZnO_2_Ag_2_Te_2_, or without long range order within the zinc oxide layers, as in Ba_2_Zn_1−*x*_O_2−*x*_Cu_2_Se_2_ described here. The majority of the *Sr*_*2*_*Mn*_*3*_*Sb*_*2*_*O*_*2*_ structured materials in the literature are reported with the *I*4/*mmm* symmetry, and have BVS values for the square planar divalent metal ion in excess of +1.6, as expected. However, there a small number which might be of interest to reinvestigate, as they are likely to contain the oxide displacement or symmetry change observed in the zinc containing layered oxychalcogenides, based on BVS values calculated from their reported structures – these are Ba_2_CuO_2_Cu_2_Se_2_ (+1.53), Ba_2_ZnO_2_Zn_2_As_2_ (+1.44) and Ba_2_NiO_2_Ag_2_Se_2_ (+1.25).^21,25^

### Optical properties and band gap

**Fig. 6:**
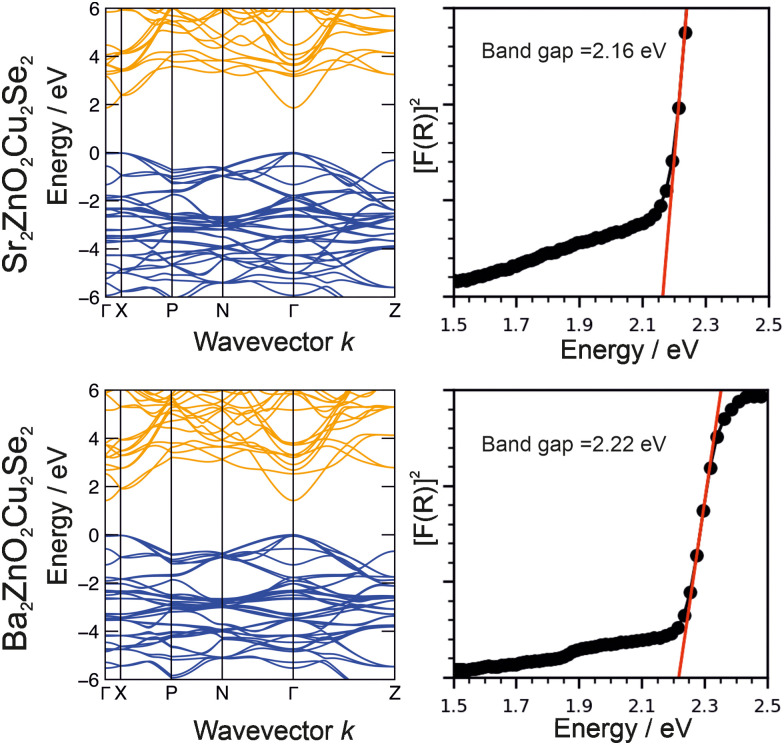
Plots of electronic band structure and optical data for Sr_2_ZnO_2_Cu_2_Se_2_ (top) and Ba_2_ZnO_2_Cu_2_Se_2_ (bottom). In each case the modelled band structure is shown on the left and on the right, plots of the of the square of the Kulbelka–Munk approximation *F*(*R*), derived from reflectance data against photon energy to determine band gaps.

The optical band gaps of Sr_2_ZnO_2_Cu_2_Se_2_ and Ba_2_Zn_1−*x*_O_2−*x*_Cu_2_Se_2_ were experimentally determined from analysis of diffuse reflectance spectroscopic data using the method described by Poeppelmeier.^43^ In this method the pseudo-absorption function *F*(*R*) is calculated using the Kubelka–Munk approximation from the diffuse reflectance data, and then [*F*(*R*)]^2^ is plotted against the photon energy, with a tangent from the absorption edge extrapolated to the abscissa to determine the band gap, as shown in Fig. 6. We find from this analysis that Sr_2_ZnO_2_Cu_2_Se_2_ has an optical band gap of 2.16 eV while that of Ba_2_Zn_1−*x*_O_2−*x*_Cu_2_Se_2_ is 2.22 eV. The electronic band structures were determined by computational modelling (in the *I*4/*mmm* symmetry) and are also shown in Fig. 6. This found that for both materials the valence band maximum (VBM) was composed of the selenium 4p orbitals, while the conduction band minimum (CBM) was principally composed of the zinc 4s orbitals. As the band edges in both compositions are derived from the same combination of orbitals, this explains the similarity in the observed band gaps, both approximately 2.2 eV, with the barium-containing material having the slightly bigger gap due to a secondary effect where its larger *a* lattice parameter reduces the overlap of the orbitals, decreasing dispersion and leading to the band gap increase.^63^

### Transport properties

In order to determine the charge transport properties of the materials, conductivity measurements were collected on ‘pristine’ undoped samples of Sr_2_ZnO_2_Cu_2_Se_2_ and Ba_2_Zn_1−*x*_O_2−*x*_Cu_2_Se_2_, and on samples of each where substitutional acceptor-type doping of the *A* site had been attempted. The doped samples were made with either sodium or potassium as the dopant, at atom percentages of 2.5%, 5% and 16.5% relative to the copper content; that is nominal compositions of *A*_*x*_Sr_2−*x*_ZnO_2_Cu_2_Se_2_ and *A*_*x*_Ba_2−*x*_ZnO_2_Cu_2_Se_2_ for both *A* = Na and *A* = K, with *x* equal to 0.05, 0.10 and 0.33, giving a total of 12 target compositions in addition to the pristine samples. The resulting samples were investigated using powder X-ray diffraction, and Rietveld refinement against the data found that in all cases the doped samples contained higher levels of impurity than the pristine samples, with a trend of diminishing sample quality with increasing dopant fraction. As these impurities would lead to increasingly unreliable conductivity results, we set a purity cut-off of 80% by mass of the target phase for inclusion in our analysis, as determined from the Rietveld refinement. For the samples with purity levels above this cut-off, four-point probe resistance measurements were carried out on annealed 8 mm pellets, and optical reflectance measurements collected to determine the sample band gaps.

For the pristine sample of Sr_2_ZnO_2_Cu_2_Se_2_ a conductivity of 0.17 S cm^−1^ was recorded. Attempts to potassium dope the sample were unsuccessful with the sample purity falling below 70% for all dopant levels, with the major impurity phases being Cu, ZnO and SrSe. The attempts at sodium doping were more successful, with purity for nominal compositions of Na_*x*_Sr_2−*x*_ZnO_2_Cu_2_Se_2_ where *x* was 0.05, 0.10 and 0.33 found to be 90.9%, 84.4% and 86.8%, respectively, with the impurities being SrSe and ZnO. Compared to the pristine sample the conductivity of the first two doped samples was found to increase by over an order of magnitude to 4.2 S cm^−1^ and 8.3 S cm^−1^ for the sodium doping fractions of *x* = 0.05 and *x* = 0.10, before dropping below the conductivity of the undoped sample to 4.5 × 10^−2^ S cm^−1^ for the *x* = 0.33 sample. No significant change in either the lattice parameters or the band gap for the doped samples was observed beyond the expected sampling error range, indicating that there was not sufficient acceptor site concentration to generate a measurable Moss–Burstein shift.

The pristine sample of Ba_2_ZnO_2_Cu_2_Se_2_ had a lower conductivity than the strontium containing analogue with a value 1.1 × 10^−3^ S cm^−1^. Attempts at sodium doping produced relatively impure samples of 84.4%, 83.7% and 78.2% for *x* = 0.05, 0.10 and 0.33 respectively, with the principal impurities being ZnO and BaCu_2_Se_2_. As the *x* = 0.33 sample was below the purity cut-off, conductivity measurements were not carried out. For the two lesser-doped samples, improved conductivity was observed compared to the pristine sample, with values of 2.2 × 10^−2^ S cm^−1^ and 5.1 × 10^−2^ S cm^−1^ for *x* = 0.05 and 0.10, an increase of just over an order of magnitude. Doping Ba_2_ZnO_2_Cu_2_Se_2_ with potassium at the lower concentrations gave much purer samples, with the main phase comprising 93.0% and 98.2% by mass for the *x* = 0.05 and 0.10 samples respectively, with only small amounts of ZnO and other minor impurities being observed in the diffraction data. These two potassium-doped samples also had improved conductivity over the pristine material, with values of 5.9 × 10^−2^ S cm^−1^ and 9.8 × 10^−2^ S cm^−1^ for the *x* = 0.05 and 0.10 samples respectively. The most heavily doped sample with nominal composition K_0.33_Ba_1.67_ZnO_2_Cu_2_Se_2_ was found to be much less pure, 75.4%, and below our cut-off. As with the strontium-containing materials, none of the doped Ba_2_ZnO_2_Cu_2_Se_2_ samples had significant differences in either cell volume or band gap compared to the pristine material.

**Fig. 7:**
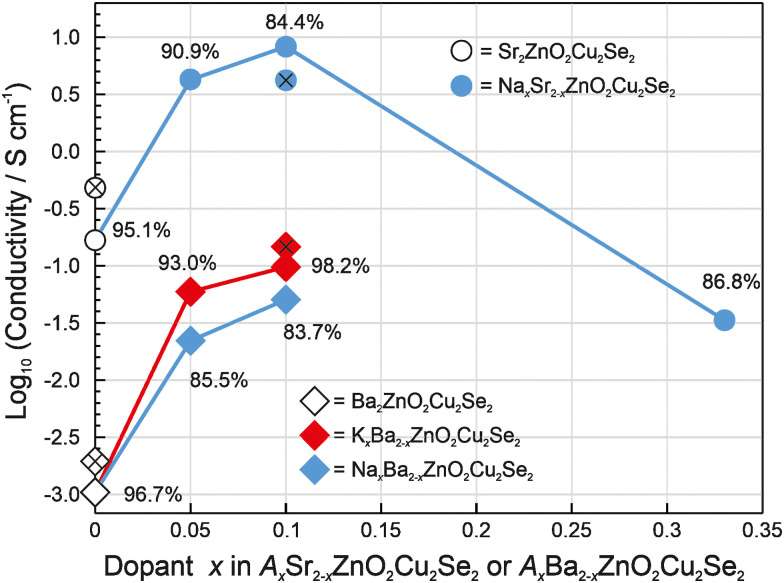
Plot of the log conductivty of pristine and doped samples of *A*_*x*_Sr_2−*x*_ZnO_2_Cu_2_Se_2_ (indicated by circles) and *A*_*x*_Ba_2−*x*_ZnO_2_Cu_2_Se_2_ (marked with diamonds). Percentage values are the sample purity. Sodium-doped samples are shown in blue, potassium-doped in red, and pristine as unfilled shapes. Hall measurements are marked with an ‘*X*’, all other values are from room temperature 4-point probe measurements.

The conductivity testing results are summarised in Fig. 7, and are tabulated alongside the pellet density, cell volume and band gap values in Table 3. Diffraction data for the samples used in the transport measurements can be found in the ESI.† From Fig. 7 we can observe the general trend of purity reducing with increased nominal doping, but with the conductivity increasing up to a peak at *x* = 0.10 (5 at% relative to the copper content). This mirrors previous work on doping of the related compound Sr_2_ZnO_2_Cu_2_S_2_ where the most conductive sample was Na_0.1_Sr_1.9_ZnO_2_Cu_2_S_2_.^16^ This indicates there is a balance between the need to reduce the electron count to increase the hole carrier concentration, and the increasing levels of impurity and defects present in the doped samples which will lower the mobility within the pellet. Based on this data, 5% of the alkali metal dopant seems to be optimal for these layered zinc compounds. The only viable choice of dopant for Sr_2_ZnO_2_Cu_2_Se_2_ is sodium, as attempts with potassium gave samples with very high levels of impurity, and while Ba_2_ZnO_2_Cu_2_Se_2_ could be doped with either Na or K without the impurity level breaching our 20% limit, the potassium doping gave both purer samples and more conductive pellets. This is likely due to the better size match between the smaller pair of ions, Na and Sr, and the larger pair, K and Ba.

**Table 3:** Experimental data from samples prepared for transport property testing. Including cell volume and purity (determined from Rietveld refinement of PXRD data) conductivity (room temperature four-point probe), band gap (UV-vis spectra) and density (percentage of theoretical density)

Sample/nominal composition	Cell volume/Å^3^	Purity (**%**)	Conductivity/S cm^−1^	Band gap/eV	Pellet density (**%**)
**Sr**_**2**_ **ZnO**_**2**_ **Cu**_**2**_ **Se**_**2**_	**304.1**	**95.1**	**1.7 × 10** ^**−1**^	**2.16**	**80.4**
Na_0.05_Sr_1.95_ZnO_2_Cu_2_Se_2_	304.0	90.9	4.2	2.18	79.0
Na_0.1_Sr_1.9_ZnO_2_Cu_2_Se_2_	303.7	84.4	8.3	2.18	85.2
Na_0.33_Sr_1.67_ZnO_2_Cu_2_Se_2_	303.9	86.8	3.4 × 10^−2^	2.16	76.5
**Ba**_**2**_ **ZnO**_**2**_ **Cu**_**2**_ **Se**_**2**_	**337.2**	**96.7**	**1.1 × 10** ^**−3**^	**2.22**	**85.1**
Na_0.05_Ba_1.95_ZnO_2_Cu_2_Se_2_	338.1	85.5	2.2 × 10^−2^	2.22	90.1
Na_0.10_Ba_1.9_ZnO_2_Cu_2_Se_2_	338.4	83.7	5.1 × 10^−2^	2.22	89.9
K_0.05_Ba_1.95_ZnO_2_Cu_2_Se_2_	337.4	93.0	5.9 × 10^−2^	2.24	79.4
K_0.10_Ba_1.9_ZnO_2_Cu_2_Se_2_	337.3	98.2	9.8 × 10^−2^	2.24	92.1

After conducting this preliminary investigation of the conductivity, Hall and Seebeck measurements were carried out on pellets of the pristine materials and the two most conductive doped samples of each material, K_0.1_Ba_1.9_ZnO_2_Cu_2_Se_2_ and Na_0.1_Sr_1.9_ZnO_2_Cu_2_Se_2_, in order to determine the carrier concentration and mobility. For pristine samples the conductivities were found to be 1.96(4) × 10^−3^ S cm^−1^ for Ba_2_ZnO_2_Cu_2_Se_2_, and 4.81(2) × 10^−1^ S cm^−1^ for Sr_2_ZnO_2_Cu_2_Se_2_, while their respective potassium and sodium doped samples had conductivity values of 1.50(1) × 10^−1^ S cm^−1^ and 4.20(2) S cm^−1^. These values follow the same trend as determined from the initial four-point technique, with all values being within one order of magnitude of the initial measurement as can be seen in Fig. 7, marked with an *X*. The Seebeck measurements confirm that these materials are p-type conductors with holes as the majority charge carriers, as positive Seebeck coefficients were measured. The hole mobilities could also be determined and were found to be similar across all the samples measured, ranging from 0.50–0.60 cm^2^ V^−1^ s^−1^, with the key difference being the carrier concentrations. For undoped Ba_2_ZnO_2_Cu_2_Se_2_ the carrier concentration was 2.2(1) × 10^16^ cm^−3^ rising to 2.2(4) × 10^18^ cm^−3^ in the alkali-metal-doped material. Undoped Sr_2_ZnO_2_Cu_2_Se_2_ has an even higher hole concentration, 6(1) × 10^18^ cm^−3^, which doping increased to 1.5(7) × 10^20^ cm^−3^. In both cases, the optimum dopant concentration increases the carrier concentration by approximately two orders of magnitude, albeit from a lower starting point for Ba_2_ZnO_2_Cu_2_Se_2_. Based on these values it is possible to determine the hole yield of the dopant, which for the sodium dopant in Sr_2_ZnO_2_Cu_2_Se_2_ is 22.8%, while that of potassium in Ba_2_ZnO_2_Cu_2_Se_2_ is 0.4%. Computational methods were also used to predict the transport properties, and these results can be seen in Table 3 alongside the Hall and Seebeck measurement results. The calculated hole masses are 0.42 *m*_e_ and 0.44 *m*_e_ for Sr_2_ZnO_2_Cu_2_Se_2_ and Ba_2_ZnO_2_Cu_2_Se_2_ respectively, with both predicted to have conductivities of ∼6 S cm^−1^ at a dopant concentration of 10^18^ cm^−3^ and ∼520 S cm^−1^ at 10^20^ cm^−3^, with carrier mobilities of between 42 cm^2^ V^−1^ s^−1^ and 32 cm^2^ V^−1^ s^−1^ (Table 4).

**Table 4:** Conductivities, carrier mobility, carrier concentration and Seebeck coefficient determined experimentally for pristine and doped samples, and the computationally predicted hole mass, and conductivity and mobility at two different carrier concentrations

	Pristine Ba_2_ZnO_2_Cu_2_Se_2_	K_0.1_Ba_1.9_ZnO_2_Cu_2_Se_2_	Pristine Sr_2_ZnO_2_Cu_2_Se_2_	Na_0.1_Sr_1.9_ZnO_2_Cu_2_Se_2_
Experimental results				
Conductivity (S cm^−1^)	1.96(4) × 10^−3^	1.50(1) × 10^−1^	4.81(2) × 10^−1^	4.20(2)
Mobility (cm^2^ V^−1^ s^−1^)	0.57(3)	0.5(1)	0.6(1)	0.6(3)
Carrier con. (cm^−3^)	2.2(1) × 10^16^	2.2(4) × 10^18^	6(1) × 10^18^	1.5(7) × 10^20^
Seebeck coefficient (μV K^−1^)	+310(40)	+140(8)	+15(8)	+9(1)

Computational results				
Hole mass/*m*_e_	0.44	N/A	0.42	N/A
Conductivity@10^18^ cm^−3^ (S cm^−1^)	6.3	N/A	6.7	N/A
Mobility@10^18^ cm^−3^ (cm^2^ V^−1^ s^−1^)	40	N/A	42	N/A
Conductivity@10^20^ cm^−3^ (S cm^−1^)	518	N/A	520	N/A
Mobility@10^20^ cm^−3^ (cm^2^ V^−1^ s^−1^)	32	N/A	33	N/A

Our most conductive sample is Na_0.1_Sr_1.9_ZnO_2_Cu_2_Se_2_ with a maximum measured conductivity value of 8.3 S cm^−1^ and carrier concentration of 1.5(7) × 10^20^ cm^−3^. This compares well with other layered oxychalcogenides in the literature, with our sample being more conductive than Na_0.1_Sr_1.9_ZnO_2_Cu_2_S_2_ (0.12 S cm^−1^), Na_0.1_Sr_1.9_GaO_3_CuS (2.2 × 10^−2^ S cm^−1^), and undoped Sr_3_Sc_2_O_5_Cu_2_S_2_ (2.8 S cm^−1^) but not as conductive as undoped LaOCuSe (24 S cm^−1^).^16,64,65^ Of particular note is the high carrier concentration achieved of approximately 10^20^ cm^−3^, as this is close to the optimal value for a transparent conductor of 10^21^ cm^−3^,^66^ achieved due to the high hole yield of the sodium dopant. However, the overall conductivity is significantly lower than the computationally predicted value of 520 S cm^−1^ for this carrier concentration. This can be understood as our measurements were collected on annealed pellets, with a measured density only 85.2% of the theoretically expected value. The experimentally measured mobility of 0.6(3) cm^2^ V^−1^ s^−1^ is much lower than the computationally predicted value of 32 cm^2^ V^−1^ s^−1^ and this indicates that the grain boundary and void effects of our low-density pellet are limiting the measured conductivity through suppression of mobility. These voids have been visualised using SEM collected on pellets of K_0.1_Ba_1.9_ZnO_2_Cu_2_Se_2_ and Na_0.1_Sr_1.9_ZnO_2_Cu_2_Se_2_ (ESI,† Fig. S14 and S15 respectively) which show that the particles vary in size from 1–10 microns, and that the cold pressing and annealing process only imperfectly compresses the particles, with a considerable fraction of the surface displaying fissures and voids limiting the measured conductivity.

This indicates that if a higher density sample was prepared, for example by spark plasma sintering, then it may be possible to achieve the much higher conductivity predicted computationally and approach the conductivity required for commercial applications. This conjecture is supported by previous work that has shown increased conductivity can be achieved for materials in high-density pellets or sputter-coated films compared to annealed pellets of the same material.^67,68^ Within this context, our measurements show the potential of sodium-doped Sr_2_ZnO_2_Cu_2_Se_2_ as a p-type conductor. It has a band gap that allows partial transparency to visible light and the sodium dopant can efficiently introduce holes into the valence band and, based on computational measurements, could have significantly improved mobility if produced in a form with a higher particle density, allowing it to be competitive with n-type transparent conductors.

Future work should focus on single crystal growth, or deposition of thin films of Sr_2_ZnO_2_Cu_2_Se_2_, and a detailed defect chemistry analysis to understand the discrepancies between our experimental measurements and the theoretically predicted maximum conductivities. It should also be noted that the defect chemistry of layered oxyselenides can be very complex;^69,70^ the champion conductivity displayed by Mg-doped LaCuOSe cannot be explained by conventional defect chemistry behaviour or computational analysis.^71^ The disperse nature of the conduction band minima, and the not overly large magnitude of the band gap also raise the tantalising prospect of bipolar dopability, which also should be tested.

## Conclusion

Two new compounds, Ba_2_Zn_1−*x*_O_2−*x*_Cu_2_Se_2_ and Sr_2_ZnO_2_Cu_2_Se_2_, have been synthesized *via* solid state synthesis under vacuum, and their structure, electronic and optical properties determined. X-ray powder diffraction, neutron powder diffraction and single crystal X-ray diffraction data analysis confirmed the *Sr*_*2*_*Mn*_*3*_*Sb*_*2*_*O*_*2*_ structure type for both materials. Both structures can be refined in the space group *I*4/*mmm* with Sr_2_ZnO_2_Cu_2_Se_2_ adopting the ideal structure, while Ba_2_ZnO_2_Cu_2_Se_2_ is best modelled as having an oxide layer with split oxide sites consistent with a linear zinc geometry. Although, according to X-ray, neutron and electron diffraction measurements there is disorder in the oxide layer in Ba_2_Zn_1−*x*_O_2−*x*_Cu_2_Se_2_. UV-vis spectra allowed optical band gaps to be determined, finding them both to be approximately 2.2 eV, indicating that they could be used for transparent conductor applications where partial visible spectrum transmission is acceptable. It was found that both materials could undergo substitutional doping at the alkaline earth sites by alkali metal ions as acceptor dopants with optimum doping at 5 at%, with sodium being a more effective dopant in Sr_2_ZnO_2_Cu_2_Se_2_ and potassium in Ba_2_Zn_1−*x*_O_2−*x*_Cu_2_Se_2_. Higher dopant concentrations led to decreasing phase purity. Hall measurements confirmed that both pristine and doped materials were p-type conductors. Sodium doping in Sr_2_ZnO_2_Cu_2_Se_2_ showed a particularly high hole generation efficiency, in excess of 20%, allowing carrier concentrations of ∼10^20^ cm^3^ to be achieved, but with the overall conductivity limited by the carrier mobility, likely due to grain boundary and impurity effects. If these could be eliminated by improved synthesis and pellet densification leading to mobility closer to the values predicted computationally, then Na_*x*_Sr_2−*x*_ZnO_2_Cu_2_Se_2_ would be of significant interest for further investigation as a p-type material for conducting coatings.

## Data availability

The data supporting this article, including diffraction data, UV-vis spectra and ‘cif’ files are available at the University of Southampton data repository, ‘eprints’ accessible from https://doi.org/10.5258/SOTON/D3113.

## Conflicts of interest

There are no conflicts to declare.

## Supplementary Material

TC-012-D4TC02458C-s001
