# Supplementary material for: Observation and enhancement through alkali metal doping of p-type conductivity in the layered oxyselenides Sr2ZnO2Cu2Se2 and Ba2Zn1−xO2−xCu2Se2
Source: J Mater Chem C Mater. 2024 Sep 19;12(43):17574–86. doi: 10.1039/d4tc02458c (PMC11440232; doi:10.1039/d4tc02458c)
Supplement: TC-012-D4TC02458C-s001 [file TC-012-D4TC02458C-s001.pdf]

Supplementary Information (SI) for Journal of Materials Chemistry C.
This journal is © The Royal Society of Chemistry 2024

Electronic Supplementary Information

Observation and enhancement through alkali metal doping of *p*-type conductivity in the layered oxyselenides $Sr_2ZnO_2Cu_2Se_2$ and $Ba_2Zn_{1-x}O_{2-x}Cu_2Se_2$

Zahida Malik,[1] Sarah Broadley,[2] Sebastian J.C. Herkelrath,[2] Daniel W. Newbrook,[3] Liam Kemp,[1] George Rutt,[1] Zoltán A Gál,[2] Jack N. Blandy,[2] Joke Hadermann,[4] Daniel W. Davies,[5] Robert D. Smyth,[2] David O. Scanlon,[6] Ruomeng Huang,[3] Simon J Clarke,[2] Geoffrey Hyett.[1]*

[1]School of Chemistry, Faculty of Engineering and Physical Sciences, Highfield Campus, University of Southampton, Southampton, SO17 1BJ, United Kingdom
[2] Department of Chemistry, University of Oxford, Inorganic Chemistry Lab, South Parks Road, Oxford, OX1 3QR, United Kingdom
[3]School of Electronics and Computer Science, University of Southampton, Southampton, SO17 1BJ, United Kingdom
[4] Electron Microscopy for Materials Science (EMAT), University of Antwerp, Groenenborgerlaan, 171, B2020 Antwerp, Belgium
[5]Department of Chemistry, University College London, 20 Gordon Street, London WC1H 0AJ, United Kingdom
[6]School of Chemistry, University of Birmingham, Edgbaston, Birmingham B15 2TT, United Kingdom

Atom	X	Y	Z	Occupancy	U_{iso} / Å^2
Cu1	0.5	0	0.25	1	0.0287(7)
Se1	0	0	0.16790(7)	1	0.0213(6)
Sr1	0.5	0.5	0.08771(6)	1	0.0174(6)
Zn1	0	0	0	1	0.0321(11)
O1	0.5	00	0	1	0.029(3)

Final refinements of $Sr_2ZnO_2Cu_2Se_2$ assuming single site oxygen in *I4/mmm*

Table S1. Unit cell parameters for $Sr_2ZnO_2Cu_2Se_2$, refined from room temperature powder XRD in the *I*4/*mmm* space group. The lattice parameters were a = 4.06703(4) Å and c = 18.38487(19) Å. Fit parameters were found to be wRp = 4.54% and Rf2 = 4.35%, with a sample purity of 95.2%.

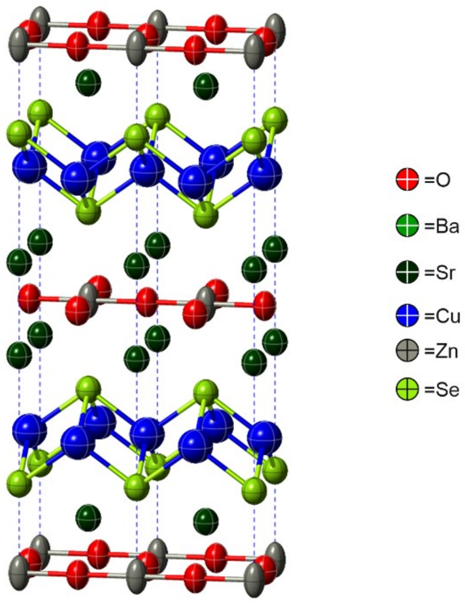

Figure S1. Representation of the unit cell of $Sr_2ZnO_2Cu_2Se_2$ derived from SXRD in the *I*4/*mmm* space group at 100 K, showing the refined anisotropic displacement ellipsoids.

					Displacement parameters / 1000 x Å^2		
Atom	*x*	*y*	*z*	occupancy	U_{11}	U_{22}	U_{33}
Cu1	0.5	0	0.75	1	9.4(3)	9.4(3)	9.0(4)
Se1	0.5	0.5	0.66865(4)	1	4.9(2)	4.9(2)	5.8(3)
Sr1	0	0	0.41193(5)	1	4.9(3)	4.9(3)	5.8(4)
Zn1	0.5	0.5	0.5	1	3.3(3)	3.3(3)	10.2(6)
O1	0.5	0	0.5	1	5(2)	4(2)	7(2)

Table S2. Unit cell parameters for $Sr_2ZnO_2Cu_2Se_2$, refined from single crystal XRD in *I*4/*mmm* space group at 100 K. Lattice parameters found to be a = 4.0562(2) Å, c = 18.2727(15) Å.

					Displacement parameters / 1000 x Å^2		
Atom	*x*	*y*	*z*	occupancy	U_{11}	U_{22}	U_{33}
Cu1	0	0.5	0.25	1	16.0(3)	16.0(3)	15.6(3)
Se1	0	0	0.16853(2)	1	6.2(3)	6.2(3)	9.8(3)

Sr1	0	0	0.41204(3)	1	6.3(2)	6.3(2)	8.7(1)
Zn1	0	0	0	1	3.5(6)	3.5(6)	21.5(3)
O1	0	0.5	0	1	8.5(5)	9.0(7)	11.1(6)

Table S3. Unit cell parameters for $Sr_2ZnO_2Cu_2Se_2$, refined from Neutron TOF powder diffraction in *I*4/*mmm* space group at 298(2) K. Lattice parameters found to be a = 4.06518(4) Å, c = 18.3751(2) Å

Initial refinements of $Ba_2ZnO_2Cu_2Se_2$ assuming single site oxygen in *I4/mmm* – later rejected

Atom	*x*	*y*	*z*	Occupancy	U_{iso} / Å^2
Cu1	0.5	0	0.25	1	0.00276(10
Se1	0	0	0.17555(10)	1	0.0163(9)
Ba1	0.5	0.5	0.09195(7)	1	0.0116(5)
Zn1	0	0	0	0.997(9)	0.0300(20)
O1	0.5	0	0	1	0.0690(60)

Table S4. Unit cell parameters for $Ba_2ZnO_2Cu_2Se_2$, refined from RT powder XRD in the *I*4/*mmm* space group. The lattice parameters were a = 4.20905(4) Å and c= 19.03416(19) Å. Fit parameters were found to be Rw_p = 3.863% and R_f^2 = 4.664%, with a sample purity of 96.8%. This provides a reasonable fit to the data – but later analysis indicates that the oxygen site is split leading to a disordered linear zinc environment.

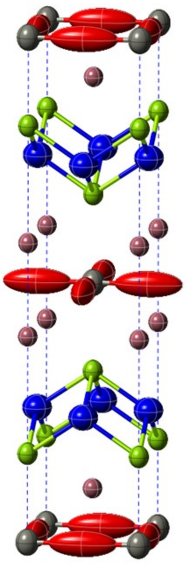

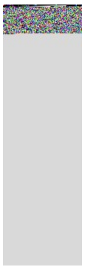

Figure S2. Representation of the unit cell of $Ba_2ZnO_2Cu_2Se_2$ derived from SXRD in the *I*4/*mmm* space group at 100 K, showing the refined elongated anisotropic displacement ellipsoids which indicate the structural distortion.

				Occupancy	Displacement parameters / 1000 x Å^2		
Atom	*x*	*y*	*z*		U_{11}	U_{22}	U_{33}
Cu1	0.5	0	0.25	1	7.87(19)	7.87(19)	6.1(3)
Se1	0.5	0.5	0.32467(2)	1	3.54(15)	3.54(15)	4.6(2)
Ba1	0	0	0.40746(2)	1	3.25(12)	3.25(12)	3.64(16)
Zn1	0.5	0.5	0.5	0.987(4)	4.9(3)	4.9(3)	6.7(4)
O1	0.5	0	0.5	1	45(2)	4.6(15)	6.1(16)

Table S5. Unit cell parameters for $Ba_2ZnO_2Cu_2Se_2$, refined from SXRD in *I*4/*mmm* space group at 100 K. Lattice parameters found to be *a* = 4.1871(2) Å, *c* = 18.9579(12) Å. Note very large U_{11} parameter.

				Occupancy	Displacement parameters / 1000 x Å^2		
Atom	*x*	*y*	*z*		U_{11}	U_{22}	U_{33}
Cu1	0	0.5	0.25	1	21.1(4)	21.1(4)	13.7(4)
Se1	0	0	0.17545(3)	1	8.04(7)	8.04(7)	10.8(5)

Ba1	0.5	0.5	0.40773(6)	1	7.3(1)	7.3(1)	4.6(8)
Zn1	0	0	0	0.931(3)	9.1(5)	9.1(5)	19.5(9)
O1	0.5	0	0	0.980(2)	7.3(5)	55.9(9)	12.1(3)

Table S6. Unit cell parameters for $Ba_2ZnO_2Cu_2Se_2$, refined from NPD in *I*4/*mmm* space group at 298 (2)K . Lattice parameters found to be a = 4.20572(5) Å, c = 19.0203(3) Å. Rw_p = 4.12%. Note very large U_{22} parameter.

				Occupancy	Displacement parameters / 1000 x Å^2		
Atom	*x*	*y*	*z*		U_{11}	U_{22}	U_{33}
Cu1	0	0.5	0.25	1	3.8(3)	3.8(3)	2.4(3)
Se1	0	0	0.17528(3)	1	2.2(3)	2.2(3)	2.6(4)
Ba1	0.5	0.5	0.40735(5)	1	1.3(3)	1.3(3)	0.0 (5)
Zn1	0	0	0	0.955(3)	1.6(5)	1.6(5)	3.8(7)
O1	0.5	0	0	0.987(3)	5.7(6)	41.6(10)	7.2(6)

Table S7. Unit cell parameters for $Ba_2ZnO_2Cu_2Se_2$, refined from NPD in *I*4/*mmm* space group at low temperature, 2 K. Lattice parameters found to be a = 4.18634(9) Å, c = 18.9683(4) Å. Rw_p = 1.91%. Note very large U_{22} parameter.

Comparison of the fit parameters for the three competing structural models for $Ba_2ZnO_2Cu_2Se_2$

As discussed in the main paper, the extended oxygen ellipsoid was indicative of a shift from square planar to linear zinc geometry. The electron diffraction data rules out both the conventional tetragonal structure and the ordered orthorhombic distortion, indicating the split site tetragonal model as the most reasonable. For completeness all three models were used with the neutron diffraction data, which confirmed that the split site also gave better or equivalent fits to the data.

Experimental temperature	Split *I*4/*mmm*	Unsplit *I*4/*mmm*	*Cmca*
2 K	1.89%	1.91%	1.88%
RT	4.06%	4.12%	4.08%

Table S8. Comparison of Rw_p fit values for the three competing models.

Final refinements of $Ba_2ZnO_2Cu_2Se_2$ modelled with split site oxygen in *I*4/*mmm*

				Occupancy	Displacement parameters / 1000 x Å^2		
Atom	*x*	*y*	*z*		U_{11}	U_{22}	U_{33}
Cu1	0.5	0	0.25	1	7.81(19)	7.81(19)	6.0(3)
Se1	0.5	0.5	0.32466(2)	1	3.48(15)	3.48(15)	4.6(2)
Ba1	0	0	0.40746(2)	1	3.41(12)	3.41(12)	3.78(16)
Zn1	0.5	0.5	0.5	0.991(4)	5.1(3)	5.1(3)	6.7(4)
O1	0.0435(11)	0.5	0.5	0.4972	5.5(8)	5.5(8)*	5.5(8)*

Table S10. Unit cell parameters for $Ba_2ZnO_2Cu_2Se_2$, refined from SXRD in *I*4/*mmm* space group at 100 K. Lattice parameters found to be *a* = 4.1871(2) Å, *c* = 18.9579(12) Å. R_1 = 1.29%, Rw2 = 2.78%, Gof = 1.104, Largest diff. peak/hole / e Å-3 0.64/-0.95.

				Occupancy	Displacement parameters / 1000 x Å^2		
Atom	*x*	*y*	*z*		U_{11}	U_{22}	U_{33}
Cu1	0	0.5	0.25	1	3.7(2)	3.7(2)	3.9(3)
Se1	0	0	0.17523(3)	1	2.6(2)	2.6(2)	4.3(3)
Ba1	0.5	0.5	0.40735(5)	1	1.4(2)	1.4(2)	1.5(4)
Zn1	0	0	0	0.958(3)	3.4(4)	3.4(4)	4.6(7)
O1	0	0.5433(4)	0	0.4874(16) / 0.5	3.9(60	1.3(6)	7.5(6)

Table S11. Unit cell parameters for $Ba_2ZnO_2Cu_2Se_2$, refined from NPD in *I*4/*mmm* space group at low temperature, 2 K. Lattice parameters found to be *a* = 4.18641(9) Å, *c* = 18.9687(4) Å. Rw_p = 1.89%

				Occupancy	Displacement parameters / 1000 x Å^2		
Atom	*x*	*y*	*z*		U_{11}	U_{22}	U_{33}
Cu1	0	0.5	0.25	1	20.2(4)	20.2(4)	14.9(5)
Se1	0	0	0.17543(3)	1	8.2(3)	8.2(3)	11.2(4)
Ba1	0	0	0.40777(5)	1	7.1(4)	7.1(4)	5.0(5)
Zn1	0	0	0	0.940(3)	9.6(6)	9.6(6)	20.7(9)
O1	0	0.5492(4)	0	0 0.4853(14) / 0.5	5.3(6)	5.3(6)	12.4(6)

Table S12. Unit cell parameters for $Ba_2ZnO_2Cu_2Se_2$, refined from NPD in *I*4/*mmm* space group at room temperature, 298 K. Lattice parameters found to be *a* = 4.20579(9) Å, *c* = 19.0207(4) Å. Rw_p = 4.06%

X-ray diffraction data and Rietveld refinement fits of doped samples of $A_xBa_{2-x}ZnO_2Cu_2Se_2$, A = Na or K

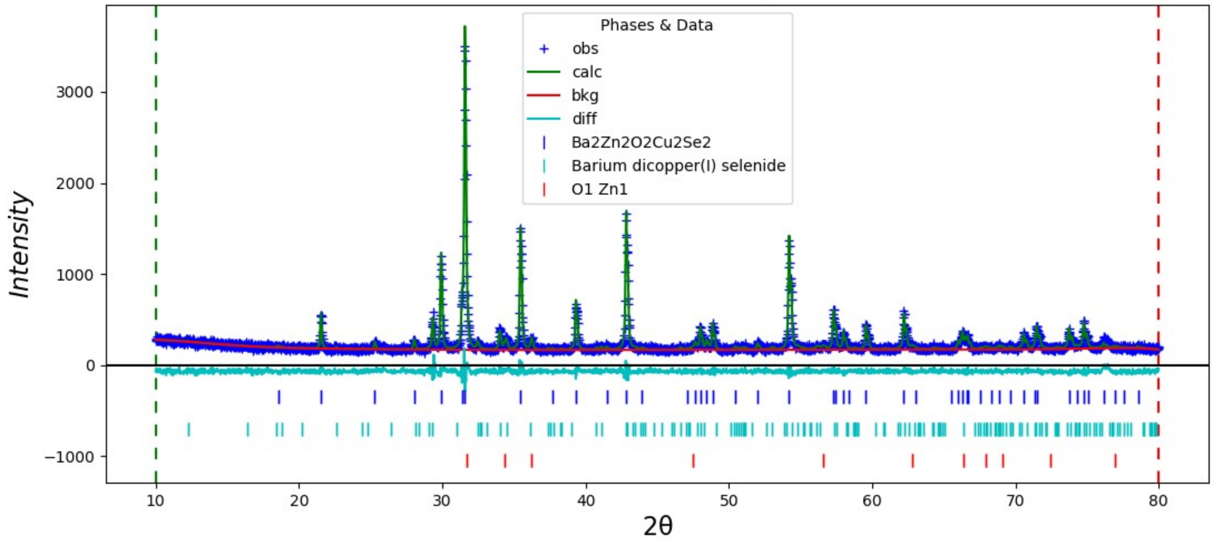

Figure S3. Rietveld refinement of attempted synthesis of $Na_{0.05}Ba_{1.95}ZnO_2Cu_2Se_2$. Sample purity of 85.5% with wRp of 6.1%.

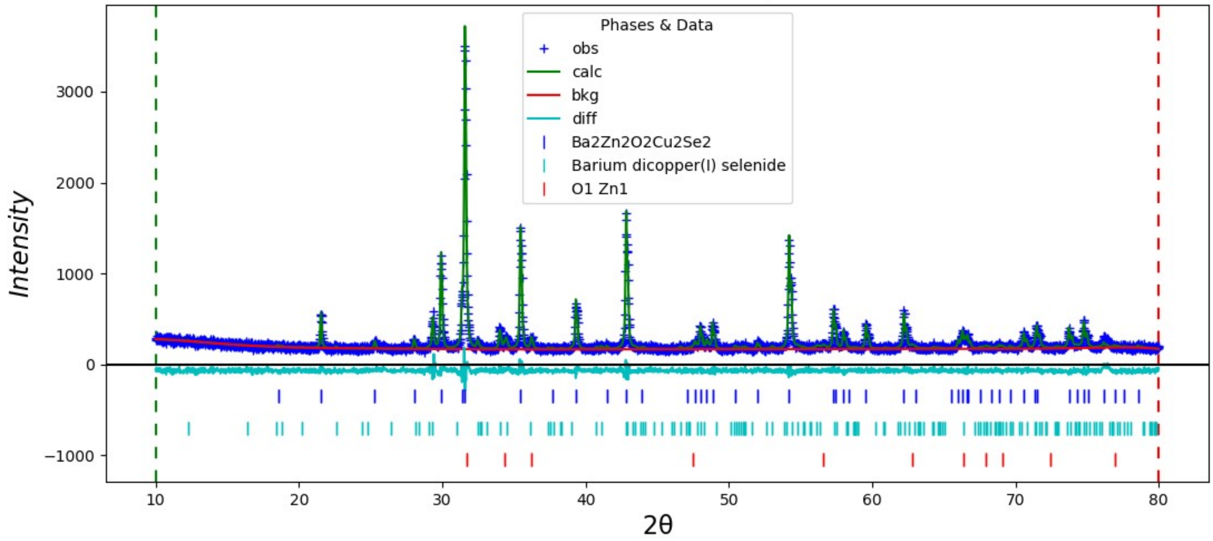

Figure S4. Rietveld refinement of attempted synthesis of $Na_{0.1}Ba_{1.9}ZnO_2Cu_2Se_2$. Sample purity of 83.7% with wRp of 7.6%.

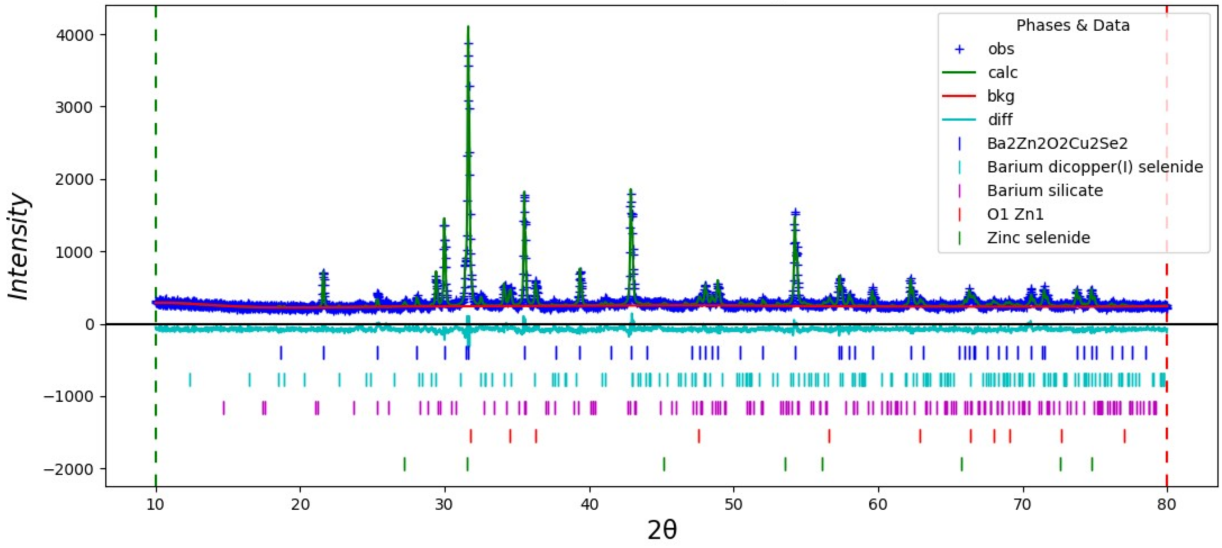

Figure S5. Rietveld refinement of attempted synthesis of $Na_{0.33}Ba_{1.67}ZnO_2Cu_2Se_2$. Sample purity of 78.2% with wRp of 7.4%

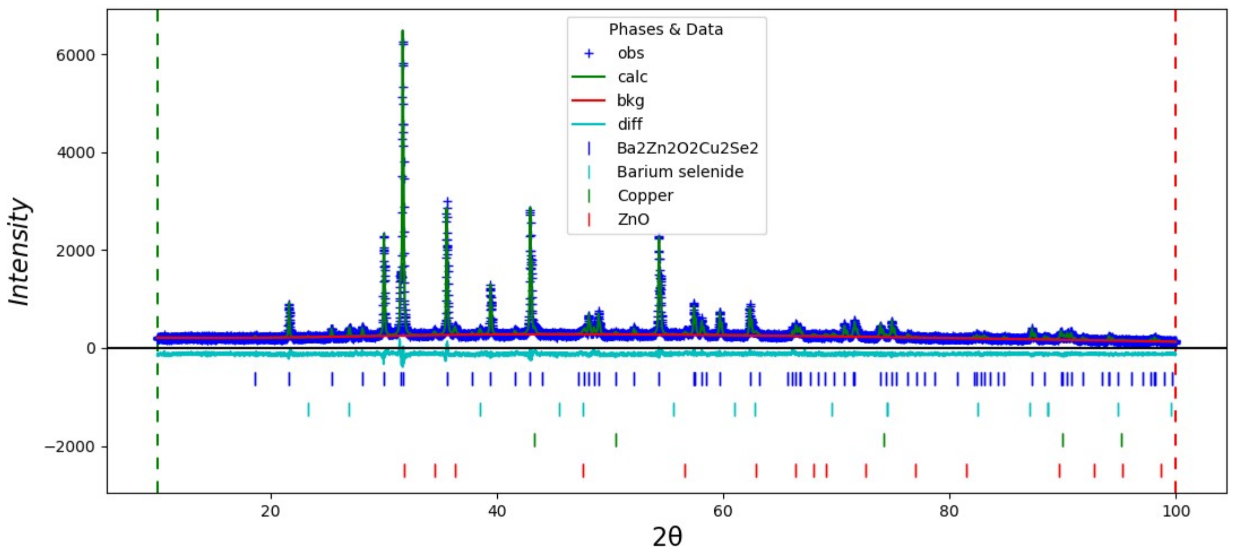

Figure S6. Rietveld refinement of attempted synthesis of $K_{0.05}Ba_{1.95}ZnO_2Cu_2Se_2$. Sample purity of 93.0% with wRp of 6.9%.

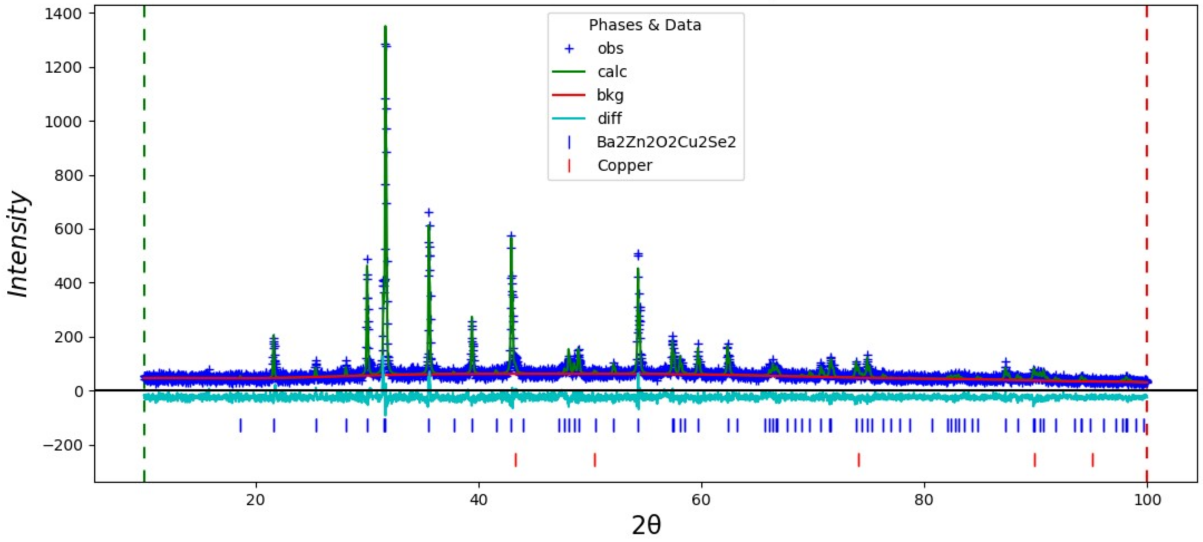

Figure S7. Rietveld refinement of attempted synthesis of $K_{0.1}Ba_{1.9}ZnO_2Cu_2Se_2$. Sample purity of 98.2% with wRp of 13.7%.

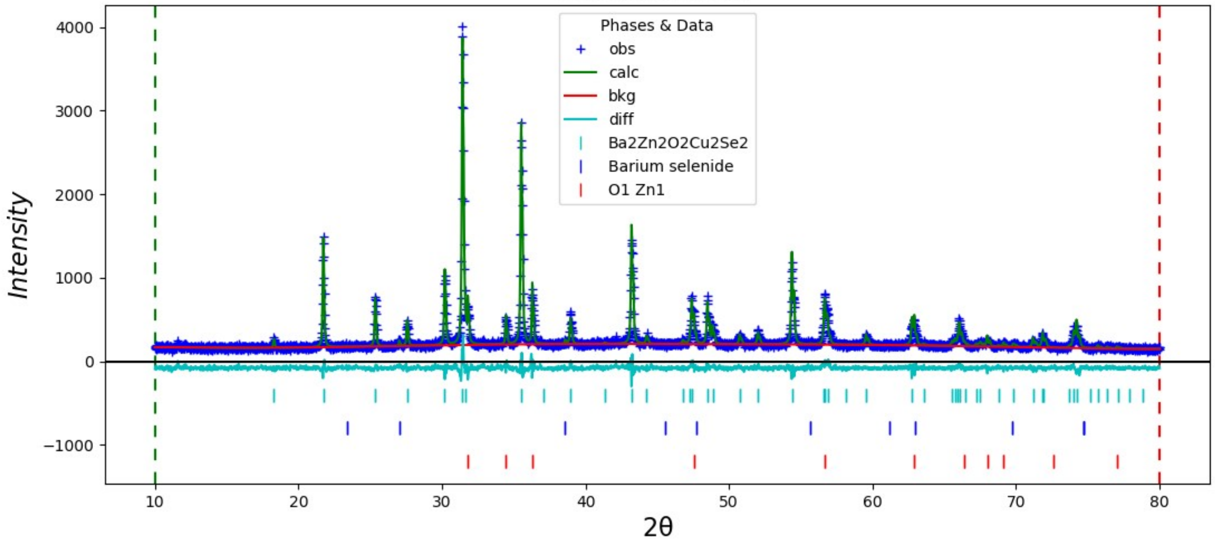

Figure S8. Rietveld refinement of attempted synthesis of $K_{0.33}Ba_{1.67}ZnO_2Cu_2Se_2$. Sample purity of 75.4% with wRp of 7.6%.

X-ray diffraction data and Rietveld refinement fits of doped samples of $A_xBa_{2-x}ZnO_2Cu_2Se_2$, A = Na or K

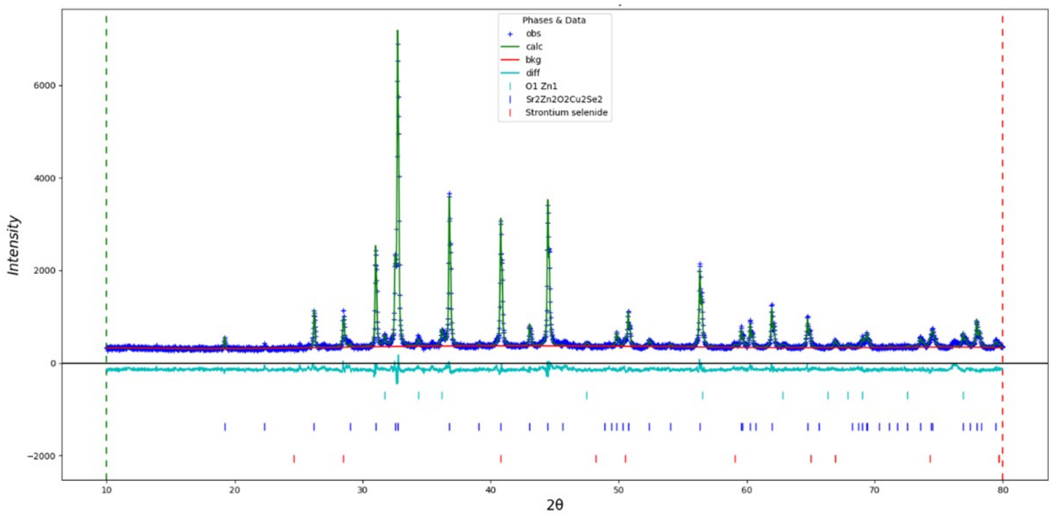

Figure S9. Rietveld refinement of attempted synthesis of $Na_{0.05}Sr_{1.95}ZnO_2Cu_2Se_2$. Sample purity of 90.9% with wRp of 6.6%.

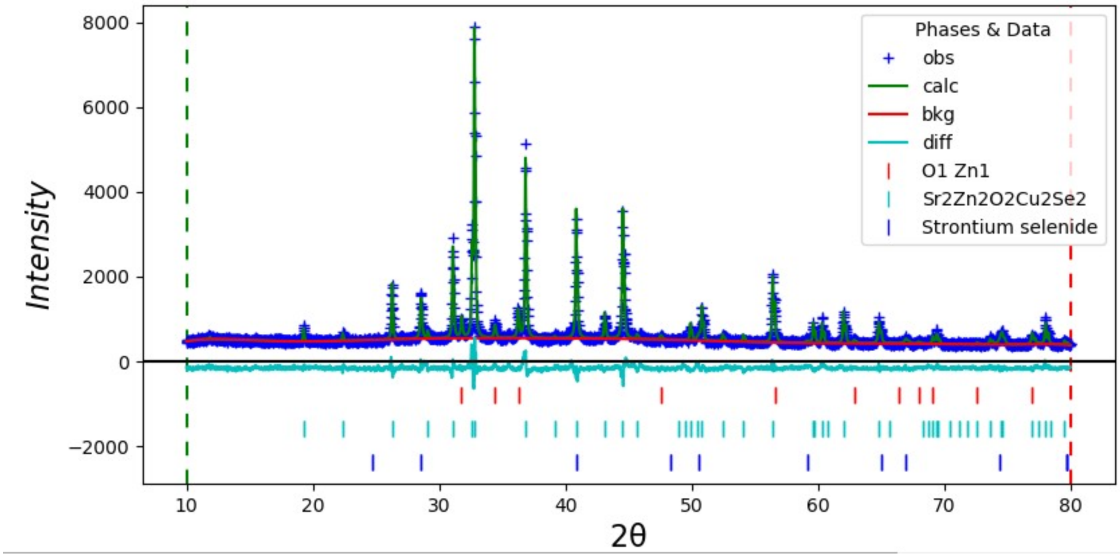

Figure S10. Rietveld refinement of attempted synthesis of $Na_{0.1}Sr_{1.9}ZnO_2Cu_2Se_2$. Sample purity of 84.4% with wRp of 7.1%.

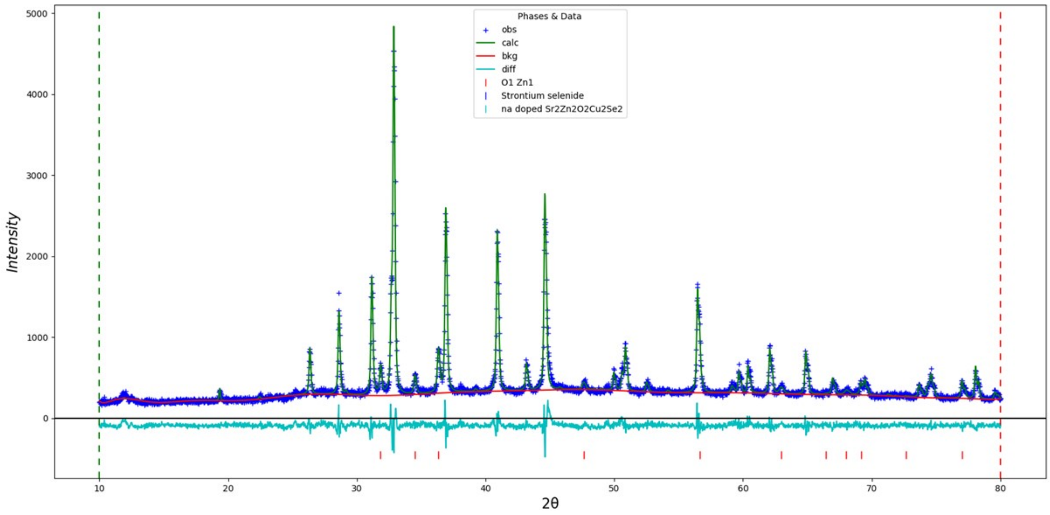

Figure S11. Rietveld refinement of attempted synthesis of $Na_{0.33}Sr_{1.67}ZnO_2Cu_2Se_2$. Sample purity of 81.5% with wRp of 7.7%.

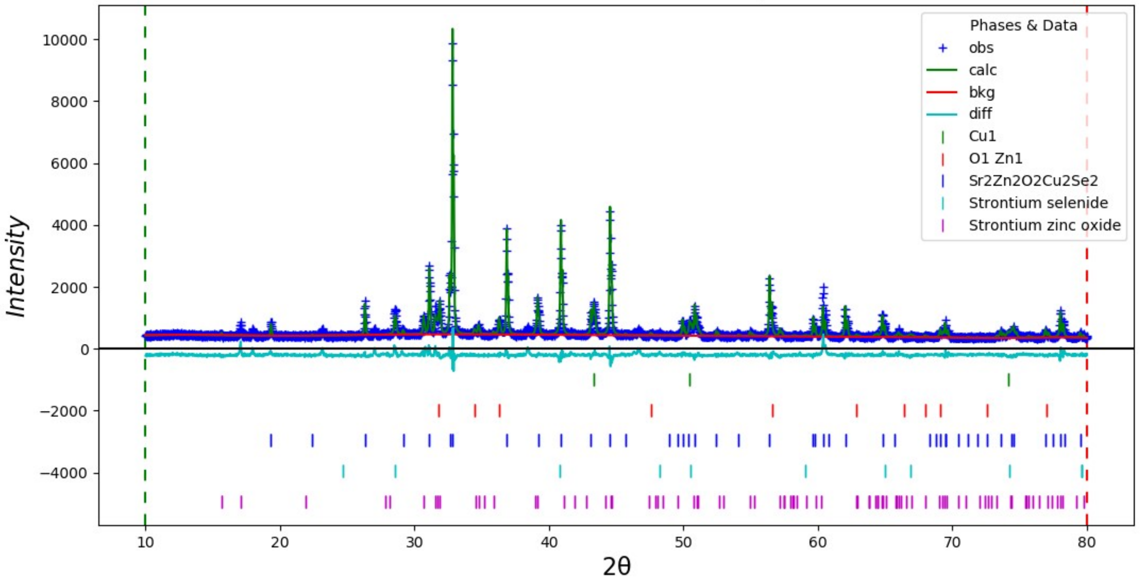

Figure S12. Rietveld refinement of attempted synthesis of $K_{0.05}Sr_{1.95}ZnO_2Cu_2Se_2$. Sample purity of 70% with wRp of 8.2%.

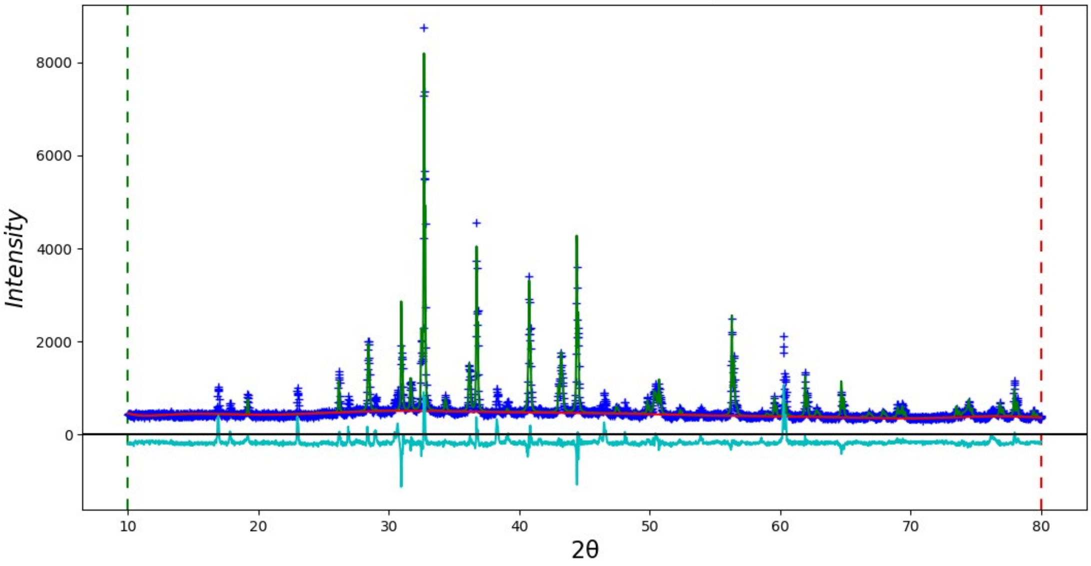

Figure S13. Rietveld refinement of attempted synthesis of $K_{0.1}Sr_{1.95}ZnO_2Cu_2Se_2$. Sample purity of 66% with wRp of 11.5%.

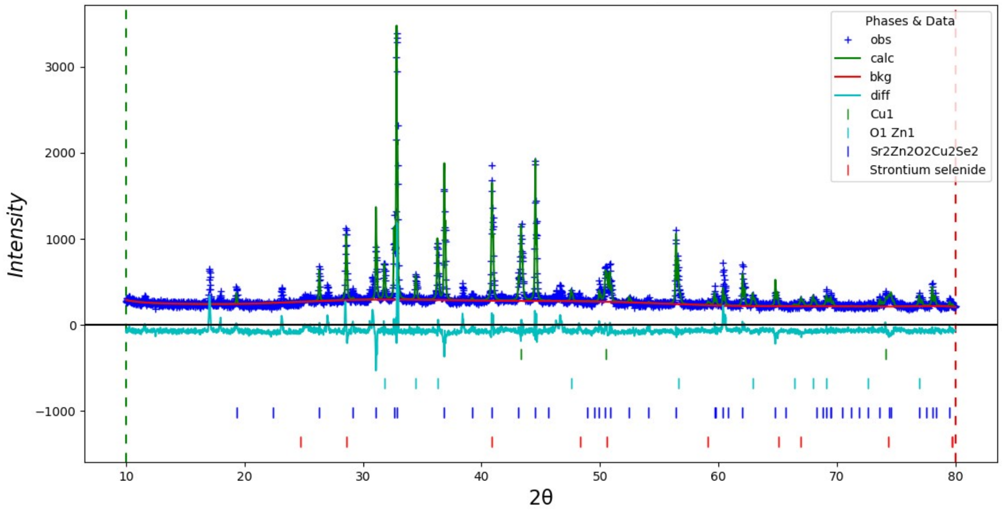

Figure S13. Rietveld refinement of attempted synthesis of $K_{0.1}Sr_{1.95}ZnO_2Cu_2Se_2$. Sample purity of 64.8% with wRp of 10.6%.

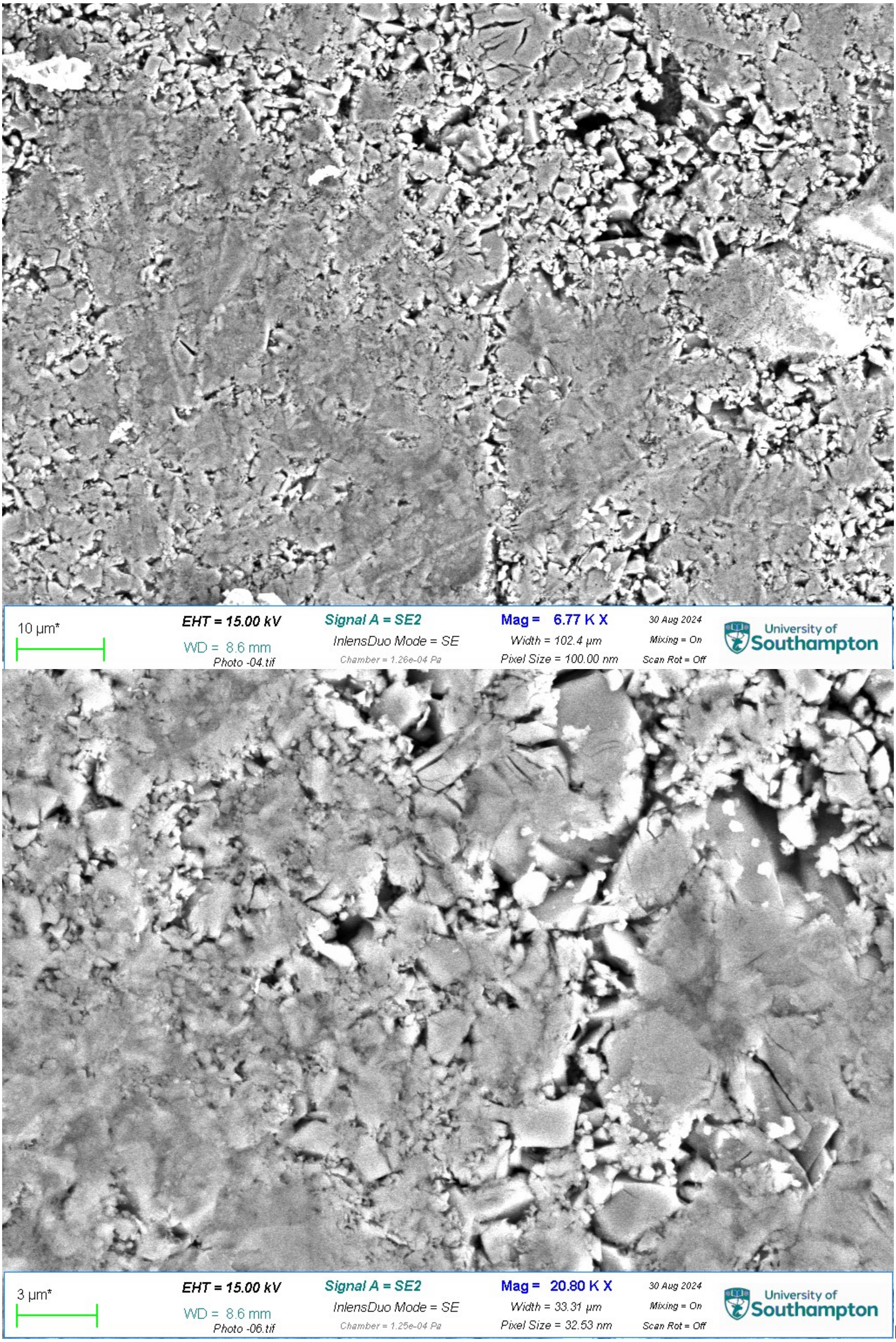

Figure S14. SEM images taken from the pellet surface of $K_{0.1}Ba_{1.9}ZnO_2Cu_2Se_2$

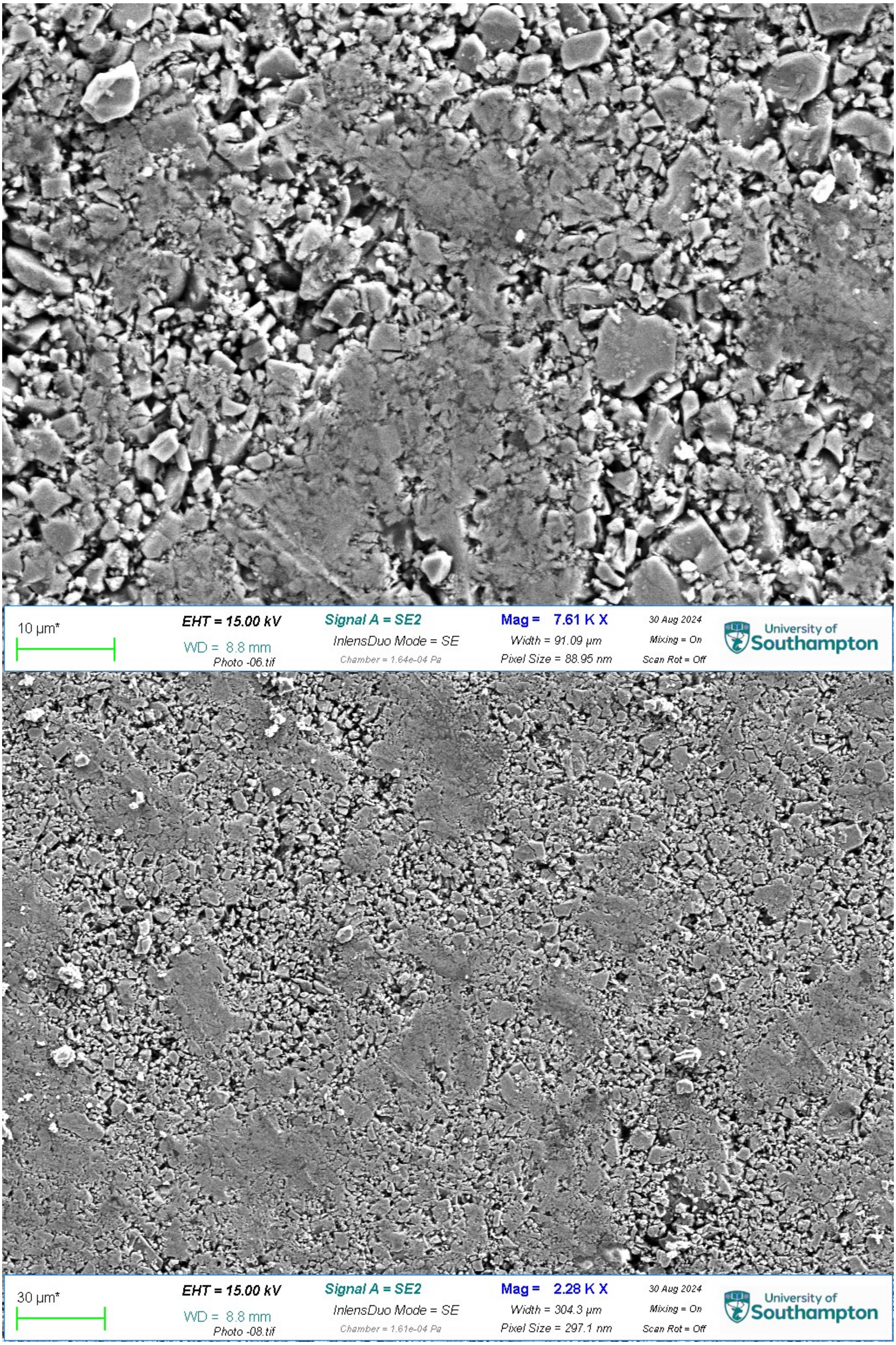

Figure S15. SEM images taken from the pellet surface of $Na_{0.1}Sr_{1.9}ZnO_2Cu_2Se_2$
